# Acetylation of Cyclic AMP Receptor Protein by Acetyl Phosphate Modulates Mycobacterial Virulence

**DOI:** 10.1128/spectrum.04002-22

**Published:** 2023-01-26

**Authors:** Yuchang Di, Siyue Xu, Mingzhe Chi, Youwei Hu, Xiao Zhang, Honghai Wang, Wenhong Zhang, Xuelian Zhang

**Affiliations:** a State Key Laboratory of Genetic Engineering, School of Life Sciences, Department of Infectious Diseases, Shanghai Key Laboratory of Infectious Diseases and Biosafety Emergency Response, National Medical Center for Infectious Diseases, Huashan Hospital, Fudan University, Shanghai, China; b Shanghai Huashen Institute of Microbes and Infections, Shanghai, China; South China Sea Institute of Oceanology

**Keywords:** cAMP receptor protein, CRP, *Mycobacterium tuberculosis*, cyclic AMP, cAMP, lysine acetylation, virulence

## Abstract

The success of Mycobacterium tuberculosis (*Mtb*) as a pathogen is partly attributed to its ability to sense and respond to dynamic host microenvironments. The cyclic AMP (cAMP) receptor protein (CRP) is closely related to the pathogenicity of *Mtb* and plays an important role in this process. However, the molecular mechanisms guiding the autoregulation and downstream target genes of CRP while *Mtb* responds to its environment are not fully understood. Here, it is demonstrated that the acetylation of conserved lysine 193 (K193) within the C-terminal DNA-binding domain of CRP reduces its DNA-binding ability and inhibits transcriptional activity. The reversible acetylation status of CRP K193 was shown to significantly affect mycobacterial growth phenotype, alter the stress response, and regulate the expression of biologically relevant genes using a CRP K193 site-specific mutation. Notably, the acetylation level of K193 decreases under CRP-activating conditions, including the presence of cAMP, low pH, high temperature, and oxidative stress, suggesting that microenvironmental signals can directly regulate CRP K193 acetylation. Both cell- and murine-based infection assays confirmed that CRP K193 is critical to the regulation of *Mtb* virulence. Furthermore, the acetylation of CRP K193 was shown to be dependent on the intracellular metabolic intermediate acetyl phosphate (AcP), and deacetylation was mediated by NAD^+^-dependent deacetylases. These findings indicate that AcP-mediated acetylation of CRP K193 decreases CRP activity and negatively regulates the pathogenicity of *Mtb*. We believe that the underlying mechanisms of cross talk between transcription, posttranslational modifications, and metabolites are a common regulatory mechanism for pathogenic bacteria.

**IMPORTANCE** Mycobacterium tuberculosis (*Mtb*) is the causative agent of tuberculosis, and the ability of *Mtb* to survive harsh host conditions has been the subject of intensive research. As a result, we explored the molecular mechanisms guiding downstream target genes of CRP when *Mtb* responds to its environment. Our study makes a contribution to the literature because we describe the role of acetylated K193 in regulating its binding affinity to target DNA and influencing the virulence of mycobacteria. We discovered that mycobacteria can regulate their pathogenicity through the reversible acetylation of CRP K193 and that this reversible acetylation is mediated by AcP and a NAD^+^-dependent deacetylase. The regulation of CRP*_Mtb_* by posttranslational modifications, at the transcriptional level, and by metabolic intermediates contribute to a better understanding of its role in the survival and pathogenicity of mycobacteria.

## INTRODUCTION

Mycobacterium tuberculosis (*Mtb*) is the causative agent of tuberculosis, and it is estimated that more than 2 billion people worldwide are infected (1). The success of *Mtb* as a pathogen is due in part to its ability to sense and respond to the dynamic and harsh environment of the host during infection, such as the low pH in macrophage phagolysosomes, nutrient deprivation, and the hypoxic center of granulomas.

The ability of *Mtb* to survive these harsh conditions has been the subject of intensive research. The differential expression of *Mtb* genes plays an important role regarding its response to various environmental stresses (2). In addition, *Mtb* can use cyclic nucleotides as second messengers to sense and respond to various external environments during an infection and then bind and alter the function of downstream effector proteins to generate a cellular response (3). Cyclic AMP (cAMP) is one such signaling molecule widely used by bacteria and eukaryotic cells. For example, cAMP signal transduction in Escherichia coli mediates its central metabolic pathway, which is also the paradigm of cAMP-mediated gene regulation in prokaryotes (4). Class I adenylate cyclase (AC) in E. coli catalyzes the synthesis of cAMP, which transduces the signal by binding to cAMP receptor protein (CRP) and activating it as a transcription factor (5). Similarly, cAMP also plays a key role in *Mtb* gene regulation in response to the host environment. Heat shock, low pH levels, starvation, nitrosative stress, oxidative stress, or propionate in culture media can increase cAMP levels in *Mtb* (5–7). Moreover, the cAMP levels in *Mtb* and a live attenuated strain of Mycobacterium bovis (bacillus Calmette-Guérin [BCG]) recovered from macrophages were 50-fold higher than those in mycobacteria exposed to tissue culture medium alone (8). Unlike E. coli, the *Mtb* genome contains at least 15 class III AC genes (9, 10), suggesting that cAMP is a particularly useful second messenger in *Mtb*.

CRP is a conserved protein in mycobacteria, is one of the three confirmed cAMP-binding effector proteins of *Mtb* (CRP*_Mtb_*, encoded by *Rv3676*) and belongs to the CRP/fumarate and nitrate reduction (FNR) family of transcription factors (3). It differs from its E. coli homolog in that binding of cAMP to CRP*_Mtb_* does not affect its DNA binding properties (5, 11). CRP*_Mtb_* is considered a virulence-associated global regulator with a regulon of at least 100 genes. The virulence of *Mtb* H37Rv *crp* deletion mutants can be fully restored by expression of *crp* from either BCG or *Mtb*, confirming that both orthologs function similarly *in vivo*, although BCG is an attenuated vaccine strain (12, 13). Importantly, CRP*_Mtb_* activates the expression of the resuscitation promoting factor A gene (*rpfA*), a growth factor capable of resuscitating the growth of mycobacterial cultures from a stationary phase (14). CRP also regulates the expression of *whiB1* (14, 15), which encodes an essential transcription factor with a nitric oxide (NO)-sensitive, oxygen-stable iron-sulfur cluster (16). More recently, CRP was also shown to directly regulate expression of the succinate dehydrogenase (Sdh1) operon (*Rv0249c*–*Rv0247c*) by binding to two promoters upstream of *Rv0250c* and *Rv0249c*, which contributed to the central metabolism of *Mtb* (17). Alternatively, *Crp*-deficient *Mtb* was shown to have attenuated growth in culture medium, macrophages, and a mouse model of tuberculosis (TB) (12, 14). Previous studies focused on CRP*_Mtb_* regulation at the level of transcription (12–15, 18), but the molecular basis for autologous regulation of CRP activity and downstream target genes that enable *Mtb* to sense and respond to its environment is not fully understood.

Posttranslational modifications (PTM) are increasingly observed in bacteria and allow cells to respond to a changing environment by modifying existing proteins to alter their activity, stability, or interactions with other molecules (19–21). *N*_ε_-lysine acetylation is one type of PTM that is conserved between prokaryotes and eukaryotes. As a dynamic and reversible process, protein lysine acetylation has been found in almost all cellular processes, such as central metabolism, protein translation, transcriptional regulation, and pathogen virulence (22–24). Increasing evidence has shown that protein acetylation also plays an important regulatory role in mycobacteria. Our group identified the modulation of central carbon metabolism by the acetylation of isocitrate lyase in *Mtb* (25). Both Yang et al. (26) and our group (27) recently found that lysine 182 (K182) acetylation of dormancy survival regulator (DosR) is critical in the response of *Mtb* to hypoxia. We, along with one other group, have also published acetylation mass spectrometry data showing that the K193 site of CRP*_Mtb_* is acetylated (27, 28). We hypothesized that the dynamic and reversible acetylation of K193 is involved in the regulation of CRP activity when *Mtb* is faced with different microenvironments during infection. The acetylation of CRP has been observed to affect class II promoter activity in E. coli (29), but the role of acetylated CRP in bacterial physiology is not well understood.

In this study, we provide evidence that the acetylation of CRP K193 results in a loss of CRP binding to its target DNA and that the reversible acetylation status of CRP K193 regulates the transcription of its downstream genes and alters *Mtb* pathogenicity and stress response. Additionally, the acetylation of K193 is directly related to mycobacterial sensing of and responding to environmental signals. Finally, CRP K193 can be acetylated and deacetylated by acetyl phosphate (AcP) and NAD^+^-dependent deacetylases, respectively, *in vitro* and *in vivo*. The regulation of CRP*_Mtb_* by posttranslational modifications, at the transcript level, and by metabolic intermediates (such as cAMP and AcP) will contribute to a better understanding of its role in the survival and pathogenicity of mycobacteria.

## RESULTS

### K193 acetylation of CRP limits its DNA-binding ability.

**FIG 1:**
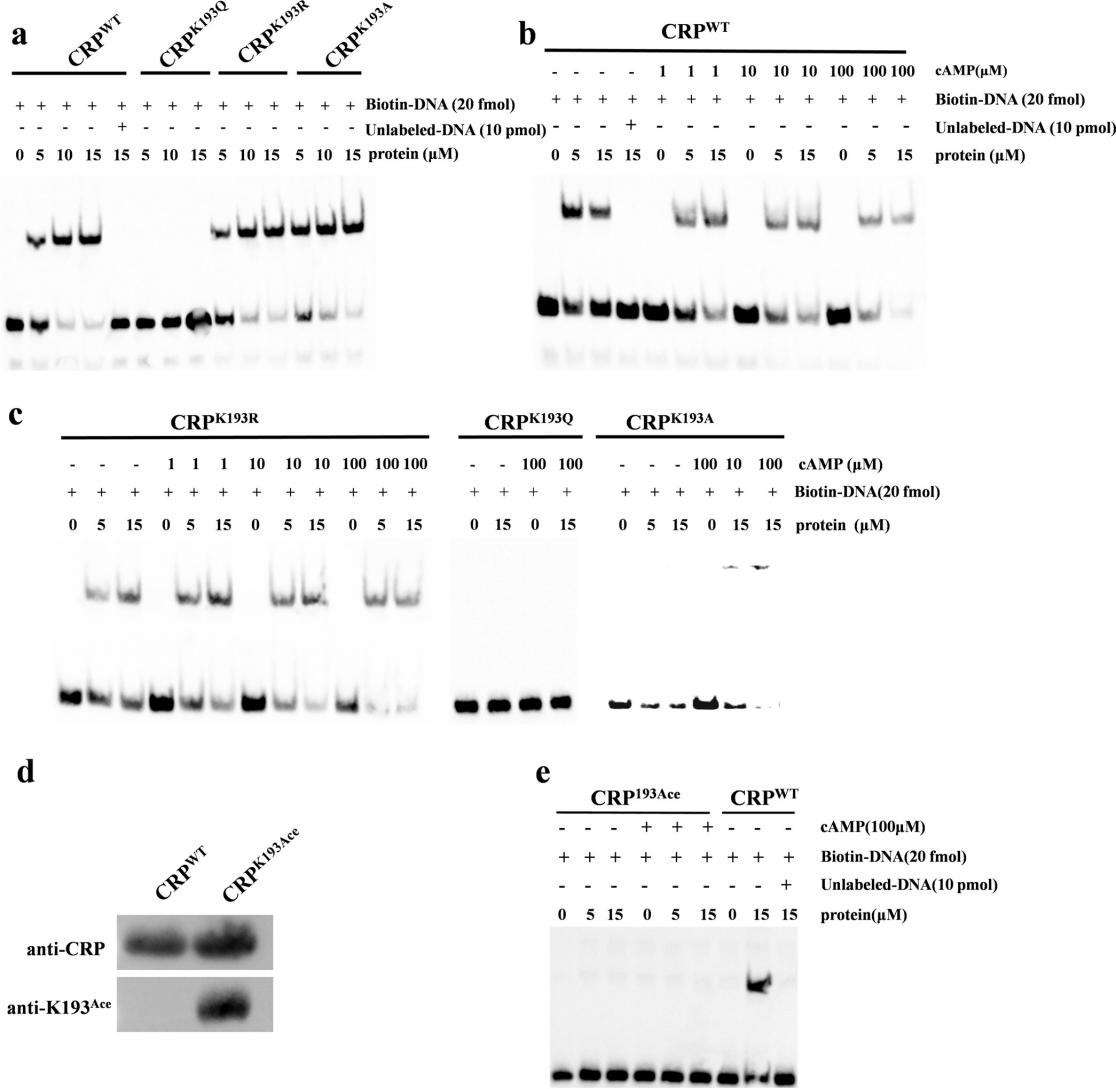
Acetylation of K193 site affects the DNA-binding ability of CRP. (a) DNA-binding abilities of CRP^WT^ and CRP mutants. EMSA was used to evaluate the DNA-binding abilities of CRP^WT^ and its derivatives at the indicated concentrations (0, 5, and 15 μM) to biotin-labeled target DNA 2D3 following a 20-min incubation. Lane 1 represents the labeled DNA alone. (b) DNA-binding abilities of CRP^WT^ were evaluated with different concentrations of cAMP. (c) Effect of cAMP on DNA-binding abilities of CRP^K193R^, CRP^K193Q^, and CRP^K193A^ evaluated by EMSA. (d) Western blot analysis of purified CRP^WT^ and CRP^K193Ace^ using a specific antibody against the acetylated K193 residue of CRP*_Mtb_*, in which K193 was successfully acetylated (CRP^K193Ace^). Antibodies specific for CRP (anti-CRP; 1:2,000) and acetylated K193 peptides (anti-K193^Ace^; 1:2,000) were used. (e) DNA-binding abilities of CRP^K193Ace^ and CRP^WT^. The binding of CRP^WT^ to the target DNA 2D3 could be completed by a 500-fold excess of unlabeled 2D3 DNA as shown in lane 5 of panel a and lane 8 of panel e. The concentrations of cAMP and proteins used in the EMSA are indicated, and each EMSA result is representative of three independent replicates. K193Q, substitution of K193 with glutamine to mimic acetylation; K193R, substitution of K193 with arginine to mimic deacetylation; K193A, substitution of K193 with glycine.

Previous acetylation mass spectrometry data revealed that the K193 residue of the CRP, which is located in the DNA binding domain, is modified by acetylation (30). Hence, we hypothesized that K193 acetylation of CRP may affect its DNA-binding affinity and influence the transcription of downstream target genes. To explore whether acetylation of K193 affected the DNA-binding ability of CRP, we expressed wild-type CRP (CRP^WT^) and K193 site-specific mutation proteins. Three DNA-binding sequences of the CRP (1D1, CCG TCT GTG AGC AAG ATC ACA TAG CT; 1B5, GTA TCT GTG ACT AAG GTC ACA GAC GC; and 2D3, CTC TAT GTG ACG AAG CCC ACA TCG AC) were synthesized and labeled with biotin (30). First, the binding affinities of CRP^WT^ proteins for the three different DNA sequences were evaluated by electrophoretic mobility shift assay (EMSA). The results showed that CRP^WT^ had the greatest affinity for 2D3 and the binding occurred in a dose-dependent manner (see Fig. S1a and b in the supplemental material). Therefore, 2D3 was used as the target DNA for subsequent EMSA experiments to evaluate the binding ability of CRP with K193 site-specific mutations. Mutations of the K193 residue had distinctive effects on ability of CRP to bind to DNA. The substitution of K193 with glutamine (CRP^K193Q^) to mimic acetylation reduced its ability to bind DNA, and increasing the protein concentration did not restore its DNA-binding capacity (Fig. 1a). However, the substitution of K193 with arginine (CRP^K193R^) to mimic nonacetylation and that with alanine (CRP^K193A^) did not have significant effects, and these CRPs bound to the target DNA in a dose-dependent manner (Fig. 1a). Since the K193A mutant was able to bind to DNA as well as the wild-type protein, it can be concluded that the lysine side chain is not necessary for the ability of CRP to bind DNA. This supports the finding that the difference in binding ability between K-to-R and K-to-Q mutants is due to the different charge of the K193 residue, similar to its acetylation status.

CRP contains a structurally conserved N-terminal cAMP-binding domain, and the affinity of CRP for DNA is differentially affected by cAMP binding. Given this information, the DNA-binding ability of the K193 mutation proteins in the presence of cAMP was further evaluated. For CRP^WT^, CRP^K193R^, or CRP^K193A^, although the shifted band intensity did not vary with cAMP or protein concentration, the loss of the unshifted band correlated with the concentrations of cAMP and CRP (Fig. 1b and c). These findings suggest that cAMP can affect the binding and shifting of CRP*_Mtb_*-DNA, but the binding under *in vitro* assay conditions may be unstable, especially in the presence of high protein concentrations. However, CRP^K193Q^, which mimics acetylated K193 CRP, lost its DNA-binding ability irrespective of cAMP levels.

To further confirm the effect of K193 acetylation on CRP function, a system of genetically encoded *N*_ε_-acetyl lysine was used to prepare a K193 site-directed acetylation protein (CRP^K193Ace^) (Fig. 1d). Consistent with the data from CRP^K193Q^, CRP^K193Ace^ lost its ability to bind target DNA, with or without cAMP (Fig. 1e). Overall, these results indicated that deacetylation of the K193 residue is critical for the DNA-binding ability of CRP.

### Acetylation of K193 regulates the transcription of target genes in mycobacteria.

**FIG 2:**
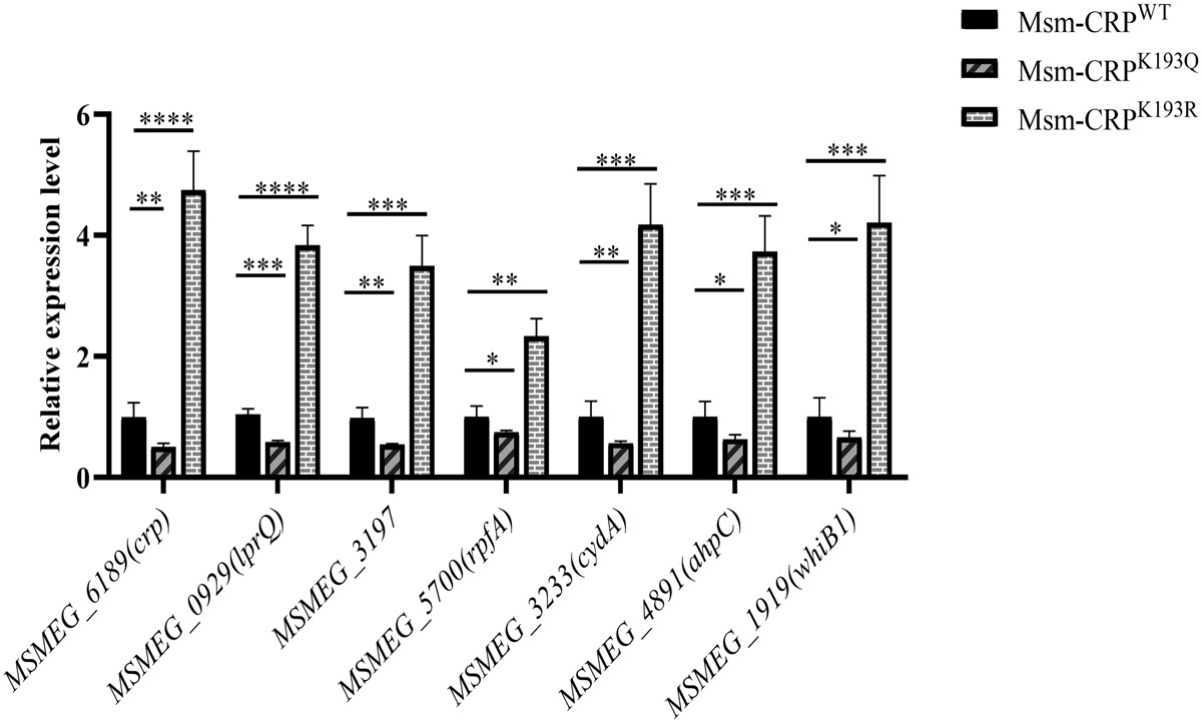
Positive charge of CRP K193 is essential for its transcriptional activity. The transcription levels of CRP*_Msm_* (*MSMEG_6189*) and its target genes in chromosome CRP variants. *MSMEG_3197* and *MSMEG_6190* in *Msm* correspond to *Rv1592c* and *Rv3677* in *Mtb*, respectively. The transcript levels were determined by qPCR and 2^−ΔΔ^*^CT^* methods. Each experiment consisted of three independent replicates. The expression of the tested gene was normalized to that of 16S rRNA and compared to that in *Msm*; significance was determined by ANOVA. *, *P* < 0.05; **, *P* < 0.01; ***, *P* < 0.001; ****, *P* < 0.0001.

Since deacetylation of the K193 residue is critical for the DNA-binding ability of CRP *in vitro*, it was next investigated whether (de)acetylation of K193 plays a key role in the physiological function of CRPs in mycobacteria. CRP is highly conserved in mycobacteria. Based on amino acid homology analysis, there are no mutations in the C-terminal DNA-binding structural domains of CRP*_Mtb_* and those of CRP from Mycobacterium marinum (CRP*_Mma_*) and Mycobacterium smegmatis (CRP*_Msm_*), and CRP*_Mtb_* differs from CRP*_Msm_* (encoded by *MSMEG_6189*) only in six amino acids located in the cAMP-binding domain (Fig. S1c). Therefore, *Msm* was selected to construct two chromosome CRP K193Q and K193R variants, designated Msm-CRP^K193Q^ (engineered CRP^K193Q^) and Msm-CRP^K193R^ (engineered CRP^K193R^). These variants were used to evaluate their effects on the transcription levels of *MSMEG_6189* itself and its target genes by quantitative PCR (qPCR) (Table 1). Compared to Msm-CRP^WT^, the Msm-CRP^K193Q^ variant, which mimics complete acetylation of K193, resulted in significantly reduced transcription (by 30 to 50%) of itself and CRP-regulated genes, whereas the Msm-CRP^K193R^ variant, which mimics a lack of K193 acetylation, had a 3- to 5-fold increase of CRP-regulated genes (Fig. 2). These data confirm the conclusion that acetylation of K193 reduces the ability of CRP to bind DNA. They also support the hypothesis that the acetylation of K193 inhibits the transcription of target genes, while its deacetylation enhances transcription in *Msm*. Furthermore, the data also suggest that a portion of CRP^WT^ is typically acetylated at baseline, which has been confirmed by acetylation mass spectrometry data for *Mtb* (27, 28).

**TABLE 1:** Target genes regulated by *MSMEG_6189* in *Msm*

*Msm* ORF	Gene product	Gene	*Mtb* ORF
*MSMEG_0929*	Conserved ErfK family lipoprotein	*lprQ*	*Rv0483*
*MSMEG_5700*	Resuscitation-promoting factor	*rpfA*	*Rv0867c*
*MSMEG_3197*	Lipase		*Rv1592c*
*MSMEG_3233*	Cytochrome *d* ubiquinol oxidase subunit	*cydA*	*Rv1623c*
*MSMEG_4891*	Alkyl hydroperoxide reductase	*ahpC*	*Rv2428*
*MSMEG_1919*	Regulatory protein	*whiB1*	*Rv3219*

### Chromosome mutants of CRP K193 affect mycobacterial growth.

**FIG 3:**
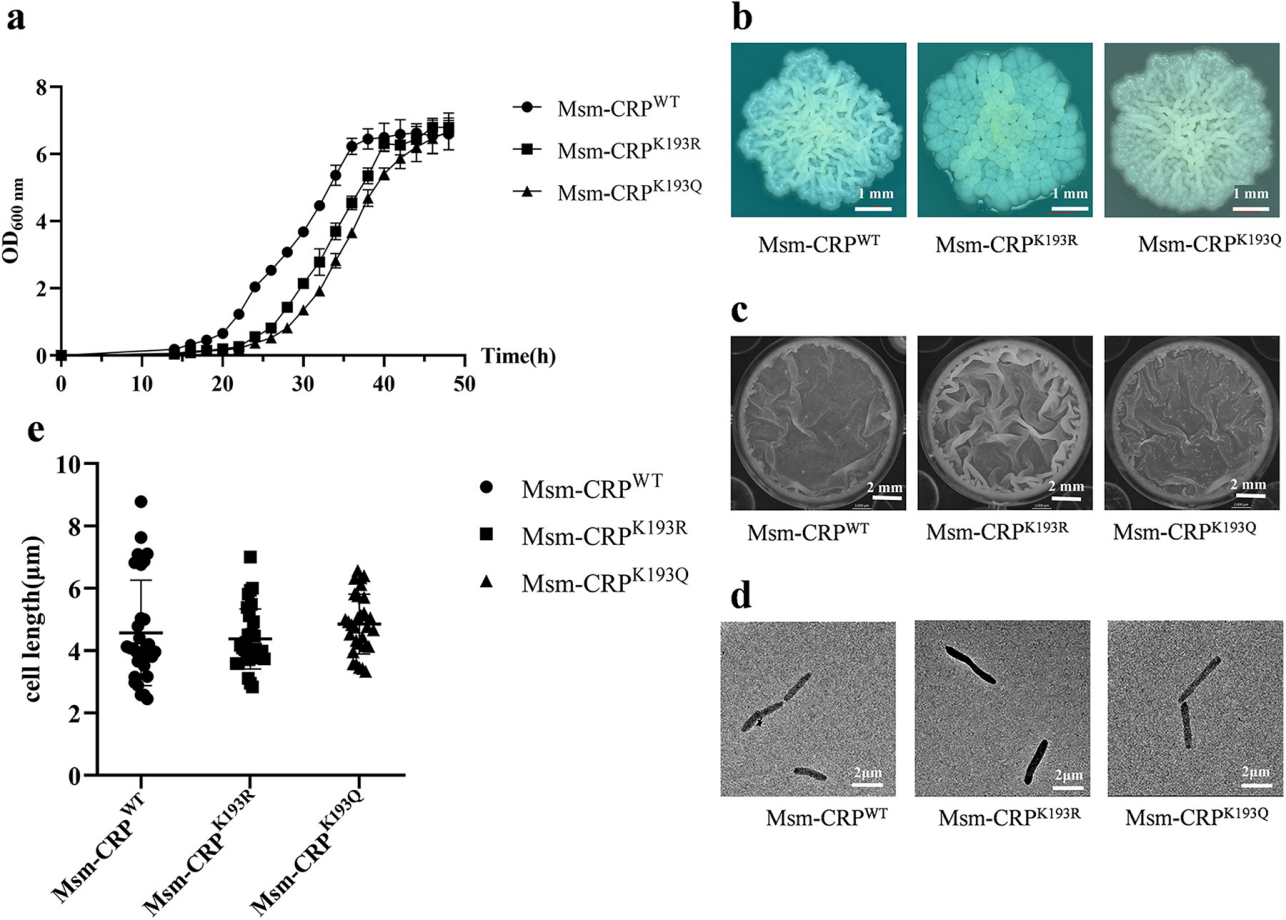
Growth phenotypes of CRP K193 site-specific mutation strains. (a) Growth curves of Msm-CRP^WT^, Msm-CRP^K193R^, and Msm-CRP^K193Q^ in 7H9-OADC medium. Data are means and standard deviations (SD) (*n* ≥ 3). (b and c) Colony morphology (b) and biofilm formation (c) of Msm-CRP^WT^, Msm-CRP^K193R^, and Msm-CRP^K193Q^. The colonies were grown in 7H10-OADC medium for 4 days at 37°C. Biofilms were grown at the air-medium interface in 12-well tissue culture plates in Sauton’s medium for 4 days at 37°C. (d) Morphological observations of *Msm*. (e) Lengths of *Msm* bacterial cells. Each experiment consisted of three independent replicates. K193R, substitution of K193 with arginine to mimic deacetylation; K193Q, substitution of K193 with glutamine to mimic acetylation.

Next, the growth curves of the Msm-CRP^K193Q^ and Msm-CRP^K193R^ mutants were assessed. The results showed that both mutants delayed the growth of *Msm* from the lag phase (Fig. 3a), with CRP^K193R^ and Msm-CRP^K193Q^ entering the stationary phase after 42 and 46 h, respectively. By comparison, Msm-CRP^WT^ required 38 h to enter the stationary phase. In addition, the colonies and pellicle biofilms of the mutants were analyzed. While Msm-CRP^WT^ had flat colonies with more folds, the Msm-CRP^K193Q^ colonies were flat with dense folds and relatively extended, and Msm-CRP^K193R^ colonies were more pleated and beaded with light yellow (Fig. 3b). Moreover, compared to Msm-CRP^WT^, which had a flat biofilm and few folds or protrusions, the biofilm of Msm-CRP^K193R^ was thicker with more folds, and the biofilm of Msm-CRP^K193Q^ was thinner with more folds and particles (Fig. 3c). The bacterial morphology and cell length were also analyzed, but no significant differences were observed for Msm-CRP^WT^ (4.57 μm), Msm-CRP^K193R^ (4.38 μm), and Msm-CRP^K193Q^ (4.85 μm) (Fig. 3d and e). Taken together, these data suggest that the K193 site of CRP and a certain level of acetylation are critical for the growth of *Msm*.

### Acetylation of K193 causes attenuated growth in high-stress environments.

**FIG 4:**
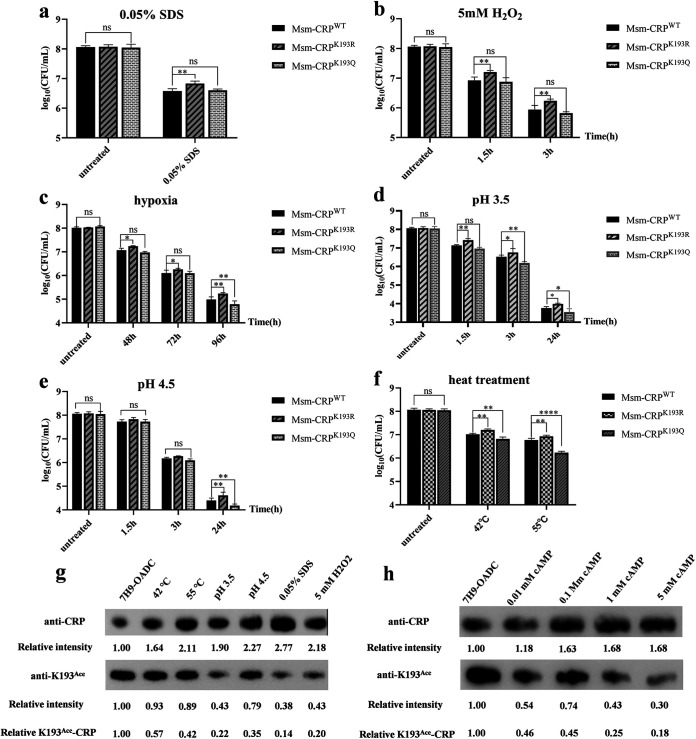
Acetylation of CRP K193 regulates the resistance of mycobacteria to external stress environments. (a to f) Survival ability of mutant strains exposed to different stress conditions, including 0.05% SDS (a), 5 mM H_2_O_2_ (b), hypoxia (c), low pHs of 3.5 (d) and 4.5 (e), and heat treatment (f). After treatment, bacteria were serially diluted 10-fold and plated on 7H10-OADC for determination of the number of CFU per milliliter. Error bars represent SD from at least three independent experiments. (g) Expression levels of CRP and acetylation levels of K193 under different conditions. The loading control for panels g and h is shown in Fig. S3a and b. (h) Expression of CRP and acetylation levels of K193 with different concentrations of cAMP. CRPs were immunoprecipitated using the anti-CRP antibody (1:2,000) and the specific anti-K193^Ace^ (1:2,000) acetylation antibody. Relative intensity represents the ratio of grayscale between different stress conditions and the normal condition (7H9-OADC). Relative K193^Ace^-CRP represents the grayscale ratio of K193 acetylation level to CRP level. Western blots were repeated independently at least three times. ns, not significant; *, *P* < 0.05; **, *P* < 0.01; ****, *P* < 0.0001.

CRP as a global regulator plays a vital role in the adaptation of bacteria to environmental changes during infection (31–33). Therefore, the survival ability of the mutant strains was assessed under different stress conditions similar to the internal environment of a macrophage, including hypoxia, low pH, oxidative stress, heat shock, and the presence of a surfactant (0.05% sodium dodecyl sulfate [SDS]), to evaluate the effect of K193 acetylation on CRP-regulated bacterial adaptation to macrophage-related stresses. The results revealed that survival of the Msm-CRP^K193R^ strain was significantly higher than that of Msm-CRP^WT^ (by approximately 0.5 log CFU [lgCFU]) when the bacterial cells were treated with different stresses (Fig. 4a to f). However, aside from treatment with H_2_O_2_ (5 mM) and SDS (0.05%), the stress resistance of Msm-CRP^K193Q^ was comparable to that of Msm-CRP^WT^, opposite to the trend observed with the Msm-CRP^K193Q^ variant. The number of surviving Msm-CRP^K193Q^ cells decreased significantly from 8.0 lgCFU to 4.7 lgCFU at low pH, 4.1 lgCFU under hypoxic conditions, and 6.2 lgCFU with heat treatment (Fig. 4c to f).

The above data focus on comparisons between the K193Q and K193R variants, since they mimic either complete acetylation or deacetylation extremes of K193. To understand the spectrum of *Msm* WT K193 acetylation, the acetylation was evaluated under different growth conditions using a specific custom antibody against the acetylated CRP*_Mtb_* K193 residue. The results showed that exposure of *Msm* to different stresses resulted in increased expression of CRP and decreased acetylation at the K193 site compared to growth in 7H9-OADC (Fig. 4g). Additionally, cAMP levels are significantly elevated in host cells infected with *Mtb* (5, 8). Therefore, the ability of cAMP to regulate the acetylation of CRP K193 in mycobacteria was evaluated. The results revealed that *Msm* treated with different concentrations of cAMP had reduced K193 acetylation levels, while the expression of CRP was increased (Fig. 4h). Taken together, the above results indicate that mycobacteria can regulate CRP activity through the reversible acetylation of CRP K193 by sensing levels of cAMP and adverse environmental conditions, which may contribute to the survival of mycobacteria in macrophages.

### Acetylation of CRP K193 reduces bacterial virulence *in vitro* and *in vivo*.

**FIG 5:**
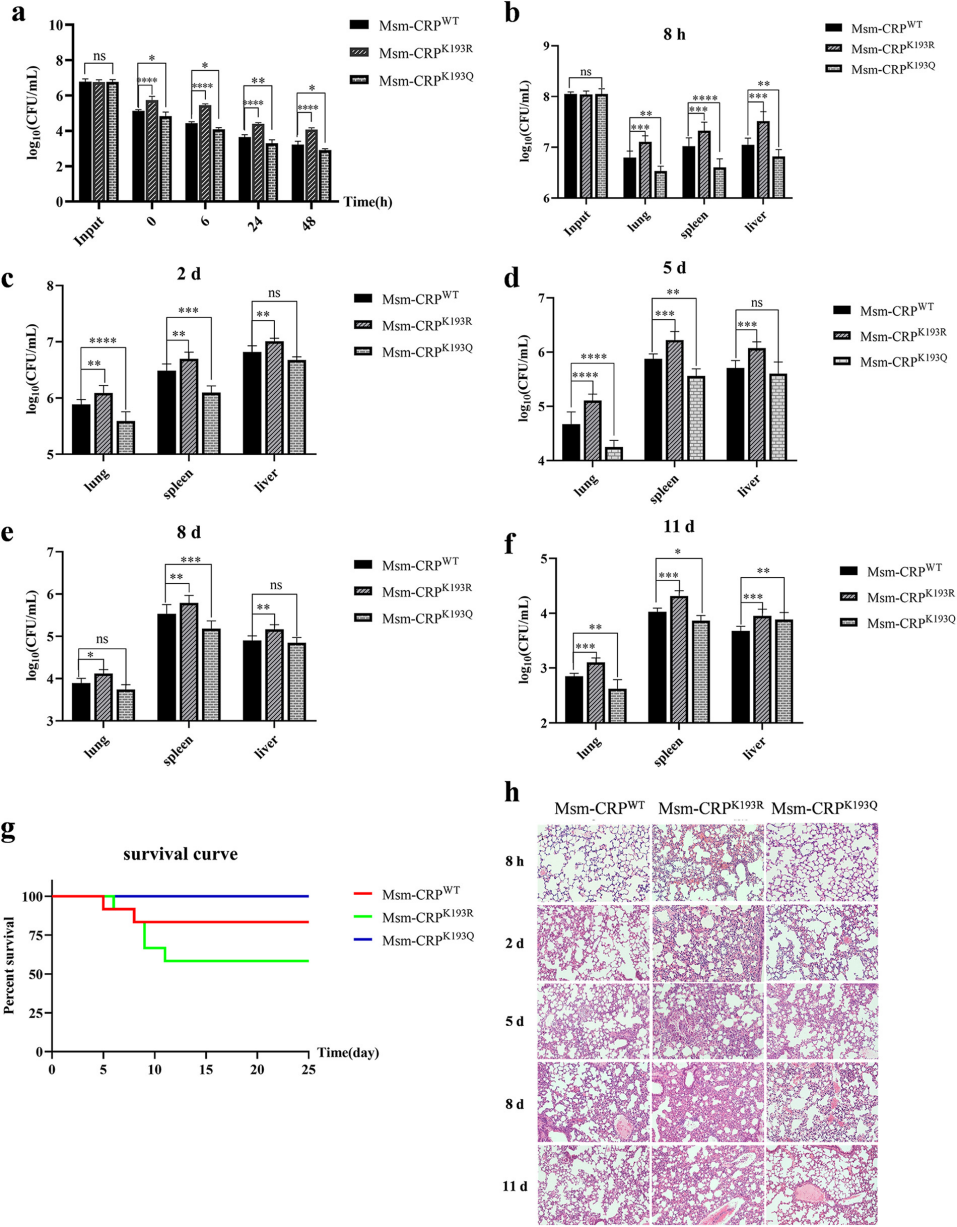
Acetylation of CRP K193 modulates bacterial virulence in mouse model. (a) Replication of bacteria in RAW264.7 cells. The net growth between 2 and 24 h was calculated from the fold change in CFU per milliliter recovered at these time points. Error bars represented SD from at least three independent experiments. (b to f) Bacterial burdens in different organs at different time points. The lungs, spleen, and liver were harvested at different time points (8 h, 2 days, 5 days, 8 days, and 11 days) after intravenous injection, and CFU were counted to quantify the number of bacteria in these organs. (g) Survival rates of mice infected by intraperitoneal injection. Eight-week-old C57BL/6 female mice were intravenously administered 1.0 × 10^8^ bacteria in 200 μL PBS. Control mice were given 200 μL PBS. The number of live mice was determined daily. (h) Hematoxylin-and-eosin-stained lungs of bacterium-treated C57BL/6 mice. ns, not significant; *, *P* < 0.05; **, *P* < 0.01; ***, *P* < 0.001; ****, *P* < 0.0001.

In the case of *Mtb*, CRP*_Mtb_* is an important transcriptional regulator strongly associated with pathogenicity (5, 14). It was hypothesized that the acetylation status of CRP K193 could regulate bacterial virulence. First, the intracellular survival ability of two CRP K193 mutants and the WT strain were evaluated. The results revealed that the invasion rates and intracellular survival rates of Msm-CRP^K193R^ significantly increased for 6 to 48 h postinfection at a multiplicity of infection (MOI) similar to that of the wild-type strain. However, Msm-CRP^K193Q^ exhibited the lowest intracellular survival ability among the three infected strains (Fig. 5a).

To further ascertain whether different levels of CRP K193 acetylation affect the virulence of mycobacteria *in vivo*, C57BL/6 mice were infected with the mutant and wild-type bacterial strains by intravenous injection. The results showed that from 8 h to 11 days after infection, the bacterial load of the Msm-CRP^K193R^ strain in the lungs, spleens, and livers of infected mice was significantly higher than that of the wild-type strain. However, the opposite results were observed in the lungs and spleens of mice infected with Msm-CRP^K193Q^; the load of Msm-CRP^K193Q^ in the liver was not significantly different from that of Msm-CRP^WT^ on days 2, 5, and 8 postinjection (Fig. 5b to f). Compared to two mice infected with Msm-CRP^WT^, which died on days 5 and 8, six mice infected with Msm-CRP^K193R^ died at the end of the 25-day experiment, whereas there were no deaths over the course of the experiment in mice infected with Msm-CRP^K193Q^ (Fig. 5g). As expected, the inflammatory infiltration and alveolar wall damage in the lung tissue of Msm-CRP^K193R^-infected mice were more pronounced than those in Msm-CRP^WT^- and Msm-CRP^K193Q^-infected mice (Fig. 5h). Taken together, these results confirmed that reversible acetylation of CRP K193 regulates the colonization and pathogenicity of mycobacteria.

### CRP K193 is acetylated by AcP.

The above results confirmed that the CRP can alter its DNA-binding affinity by reversible acetylation of K193 and thereby regulate downstream gene expression to ultimately affect the pathogenicity of mycobacteria. Therefore, the mechanism responsible for regulating the acetylation of CRP K193 in mycobacteria was evaluated.

**FIG 6:**
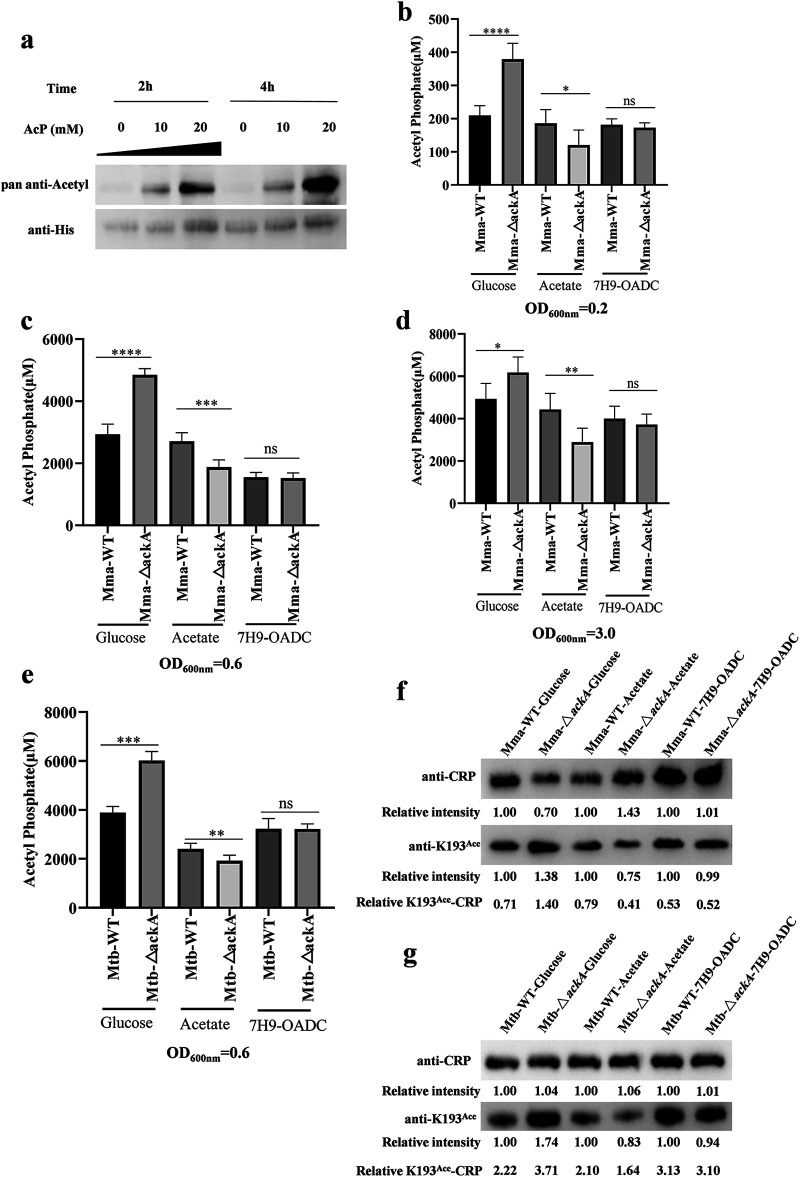
Acetylation of CRP K193 is regulated by AcP. (a) Western blotting was used to verify whether CRP can be acetylated by AcP *in vitro* with the indicated concentrations of AcP (lanes 2, 3, 5, and 6). Lane 1 represents CRP alone. CRP was immunoprecipitated using an anti-His antibody (1:5000). CRP acetylation levels were determined using the pan anti-acetyllysine antibody (1:2,000). (b to e) Intracellular concentrations of AcP at different phases of growth. *Mma* (b to d) and *Mtb* (e) cells were harvested at the indicated time points. The intracellular concentrations of AcP at early exponential phase (b), late exponential phase (c), and stationary phase (d) were measured. Error bars indicate SD from triplicate measurements. (f and g) Expression of CRP and the acetylation level of K193 in the wild-type strain and the *ackA* knockout strain with different carbon sources. *Mma* (f) and *Mtb* (g) were cultured in 7H9-AC (BSA and catalase) with different carbon source supplementation. CRP was immunoprecipitated using an anti-CRP antibody (1:2,000), and K193 acetylation level was determined using the anti-K193^Ace^ antibody (1:2,000). Relative intensity represents the ratio of grayscale between the *ackA* knockout strain and the WT with each carbon source. Relative K193^Ace^-CRP represents the grayscale ratio of K193 acetylation to CRP levels. Each experiment consisted of three independent replicates. Statistical significance was determined with Student’s *t* test. ns, not significant; *, *P* < 0.05; **, *P* < 0.01; ***, *P* < 0.001; ****, *P* < 0.0001.

The *N*_ε_-amino group of a lysine residue can be acetylated either through canonical enzymatic acetylation or nonenzymatically by AcP in bacteria (34, 35). It was reported previously that CRP acetylation is elevated when AcP levels are high in E. coli (29, 34, 35). Therefore, the modification of CRP*_Mtb_* with different concentrations of AcP was evaluated *in vitro*. The results revealed that CRP*_Mtb_* could be acetylated by AcP dose- and time-dependently, according to AcP concentration and incubation time *in vitro* (Fig. 6a). AcP is an intermediate of the phosphotransacetylase-acetate kinase (AckA) pathway (36), which is reversible. To determine whether acetylation of CRP is regulated by AcP *in vivo*, *ackA* was deleted in *Msm*, M. marinum (*Mma*-Δ*ackA*), and *Mtb* H37Ra (*Mtb*-Δ*ackA*) strains (Fig. S2). Unfortunately, the knockout *Msm* strain was not successfully obtained.

Theoretically, following the knockout of the *ackA* gene (encoding AckA), different carbon sources will produce different products according to the metabolism of AcP. First, the intracellular concentrations of AcP in two Δ*ackA* strains at different growth stages were determined using glucose and acetate as single carbon sources. As expected, compared to the wild-type strains, the AcP depletion pathway was blocked in the Δ*ackA* strains when glucose was used as a single carbon source, leading to an accumulation of AcP. The AcP synthesis pathway was blocked in the knockout strains when acetate was used as a single carbon source, and the intracellular concentration of AcP decreased. There was no significant difference in intracellular AcP concentrations when strains were cultured in 7H9 broth with oleic acid-albumin-dextrose-catalase (OADC) enrichment (Fig. 6b to e). The concentrations of AcP in mycobacteria at different growth phases were evaluated, and the intracellular AcP concentration progressively increased with the bacterial growth phase (Fig. 6b to d).

Since AcP was shown to acetylate CRP *in vitro*, the acetylation of CRP was further evaluated in *Mma-*Δ*ackA* and *Mtb*-Δ*ackA* strains under different carbon source conditions. The results shown in Fig. 6f and g indicated that the acetylation level of CRP K193 was consistent with intracellular AcP concentration in the Δ*ackA* mutants and the control strains. Compared to the control *Mma* and *Mtb* strains, and with glucose as the carbon source, higher intracellular AcP levels in the *Mma-*Δ*ackA* and *Mtb*-Δ*ackA* strains increased CRP K193 acetylation (Fig. 6f and g). In contrast, with acetate as the carbon source, the AcP levels and the acetylation of CRP K193 were decreased in the *Mma-*Δ*ackA* and *Mtb*-Δ*ackA* strains. Acetylation did not differ significantly among Δ*ackA* strains and control strains when AcP levels were steady during growth in 7H9 medium. These results support the hypothesis that AcP can act as an acetyl donor for CRP and the acetylation of CRP by AcP is a common modification mechanism used by mycobacteria.

### CRP^193Ace^ could be deacetylated by an NAD^+^-dependent deacetylase.

**FIG 7:**
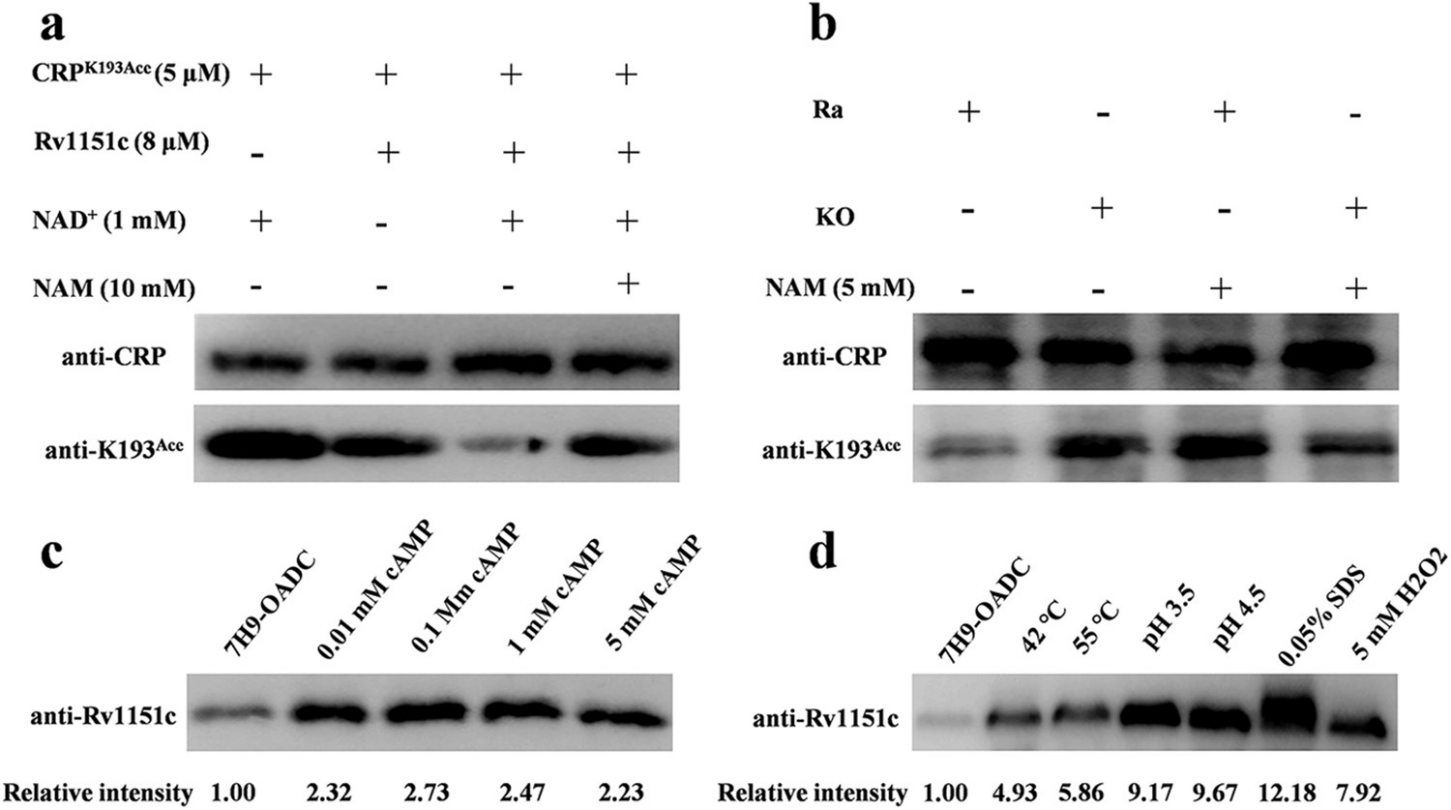
CRP K193 can be deacetylated *in vitro* and *in vivo*. (a) Rv1151c deacetylates CRP in a dose-dependent manner *in vitro*. Purified CRP^K193Ace^ (5 μM) was incubated with NAD^+^ (1 mM) and Rv1151c (8 μM) at 25°C for 2 h. The acetylation levels were evaluated by Western blotting with anti-CRP (1:2,000) and anti-K193^Ace^ (1:2,000) antibodies. (b) Acetylation levels of CRP in wild-type H37Ra (Ra) and a *MRA_1161* (*Rv1151c*, homolog in H37Ra) deletion mutant (knockout [KO]) *in vivo*. (c and d) Expression of Rv1151c under different cAMP concentrations (c) and different stress conditions (d). Cell extracts (20 μg per lane) were analyzed by Western blotting with anti-CRP (1:2,000), anti-K193^Ace^ (1:2,000), and anti-Rv1151c (1:2,000) antibodies. The loading controls for panels c and d can be found in Fig. S3c and d. Relative intensity represents the ratio of grayscale between different stress conditions and the normal condition (7H9-OADC). Results are representative of three independent experiments.

Currently, the deacetylation of acetyllysines is thought to be catalyzed by lysine deacetylase. CobB of the sirtuin family has been shown to deacetylate YfiQ-dependent acetylation and AcP-dependent acetylation in E. coli (37–40). Rv1151c (encoded by *Rv1151c*) is the only homolog of sirtuin found in the *Mtb* genome, which possesses NAD^+^-dependent deacetylase activity (27). Therefore, the regulation of CRP acetylation through deacetylation by Rv1151c was assessed. The results showed that CRP could be deacetylated by Rv1151c in a NAD^+^-dependent manner *in vitro*. The presence of nicotinamide (NAM) (10 mM), an inhibitor of NAD^+^-dependent deacetylases, prevented effective deacetylation by Rv1151c, indicating that CRP was a substrate for Rv1151c (Fig. 7a). Next, a *Rv1151c* knockout strain of H37Ra was used to determine whether CRP could be deacetylated *in vivo* in *Mtb* (25). The results showed that CRP K193 was acetylated in both the wild-type and *Rv1151c* deletion strains but that K193 acetylation levels in the mutant strain were significantly increased compared to those in the wild-type strain. The deacetylation effect on K193 CRP in the wild-type strain was inhibited by treatment with NAM (5 mM) in the culture medium (Fig. 7b), suggesting that acetylated CRP K193 can be deacetylated by an NAD^+^-dependent deacetylase both *in vitro* and *in vivo*. Since the acetylation of CRP K193 was significantly changed by different stress conditions and was downregulated by cAMP (Fig. 4g and h), the expression of Rv1151c was assessed to determine its potential regulation by cAMP levels and different stresses. It was found that deacetylase expression was significantly increased when the bacteria were exposed to different concentrations of cAMP (Fig. 7c) or different stress conditions (Fig. 7d). Taken together, these data suggest that acetylated CRP K193 can be deacetylated by an NAD^+^-dependent deacetylase both *in vitro* and *in vivo*.

## DISCUSSION

*Mtb* successfully invades host cells and has evolved multiple mechanisms to adapt to adverse conditions in the host, among which the second messenger cAMP plays a key role in gene regulation when *Mtb* responds to host-relevant conditions (2, 5). CRP, one of three cAMP effectors identified in *Mtb*, is an important global transcriptional regulator of *Mtb* during infection, especially in response to conditions of stress such as hypoxia and low pH (30). A previous study reported a widespread transcriptional alteration, covering more than one-fifth of the total *Mtb* genome (17). Although CRP*_Mtb_* plays a prominent role in cellular processes, the mechanistic basis whereby CRP regulates its own function and downstream gene expression to influence the pathogenicity of *Mtb* is not fully understood. Previous protein acetylation mass spectrometry data of mycobacteria consistently detected acetylated K193 in CRP (27, 28). In this study, the important role played by acetylated K193 in regulating its binding affinity to target DNA and influencing the virulence of mycobacteria was revealed.

The mutant of CRP K193 that mimicked complete deacetylation, Msm-CRP^K193R^, upregulated the transcription of regulatory genes by 3- to 5-fold (Fig. 2). In contrast, the Msm-CRP^K193Q^ strain, which mimicked complete K193 acetylation, resulted in reduced transcription, with a 30 to 50% downregulation compared to the CRP^WT^ strain (Fig. 2). These findings indicate that CRP^WT^ in the physiological state does not remain fully (de)acetylated but that (de)acetylation is a reversible and dynamically changing process where the target gene transcription profile likely lies between these two extremes. CRP expression was increased when the bacteria were placed in adverse environments, which was consistent with a previous study (25). Meanwhile, the acetylation level of the K193 site was significantly decreased (Fig. 4g) when mycobacteria were exposed to the different stresses, which confirmed the hypothesis that K193 acetylation is a dynamic process. In addition, the K193R and K193Q mutants both exhibited growth defects relative to the wild-type strain, supporting the hypothesis that some acetylation is ideal for mycobacterial growth.

Furthermore, CRP expression was increased and the acetylation level of K193 was decreased when mycobacteria were treated with different concentrations of cAMP (Fig. 4h). It has been reported that the activity of AC is directly affected by pH, fatty acids, and carbon dioxide (CO_2_) and the expression of AC is controlled by hypoxia and starvation in *Mtb*, which ultimately increases cAMP levels (2, 5–8). Increased cAMP in host cells during infection is a strategy that *Mtb* uses to suppress innate immune function, dampen the phagocytic response, and reduce intracellular killing (5). Therefore, we concluded that *Mtb* regulates CRP activity by increasing CRP expression, but also through the reversible acetylation of CRP K193 when sensing cAMP and unfavorable conditions of the microenvironment. This ultimately contributes to its own survival within the host. The regulation of CRP acetylation by cAMP concentration before and after macrophage infection would be accompanied by a direct effect on the interaction between CRP and DNA.

Results from the mouse infection model showed that the CRP^K193Q^ strain mimicking acetylation is less pathogenic in terms of bacterial burden in the lungs and spleen as well as by immunohistochemical assays. The survival assays also showed similar trends: six Msm-CRP^K193R^-infected mice and two Msm-CRP^WT^-infected mice died, while all Msm-CRP^K193Q^-infected mice survived the infection. Therefore, reversible K193 acetylation regulates the physiological function of CRP, which in turn alters the pathogenicity of mycobacteria *in vivo*. A similar scenario has been observed in the regulation of DosR, the well-known dormancy survival regulator in *Mtb* (26, 27). There is growing evidence that acetylation is involved in the functional regulation of transcription factors in prokaryotes, such as CheY (41, 42) and RcsB in E. coli (43), PhoP in Salmonella (44), and NrtR in *Msm* (45), suggesting that regulation of this PTM or even cross talk with other PTMs is a conserved regulatory mechanism in bacteria. The close interrelationship between transcriptional and PTM networks affects physiological consequences, reflecting a high degree of complex regulation in prokaryotes.

Given the effect of acetylation modification on mycobacterial virulence, the mechanism of CRP acetylation was explored. In general, protein acetylation can be categorized into two types: the enzymatic action of the acetyltransferase Pat and the nonenzymatic action of AcP (34, 35). AcP, the high-energy intermediate of the phosphotransacetylase-acetate kinase pathway, has been reported to be a major acetyl donor regulating global acetylation in E. coli and Bacillus subtilis (36). AcP has been shown to influence the function of RcsB (37), isocitrate dehydrogenase and malate dehydrogenase (46, 47), ACs (48), topoisomerase (49), and enolase (23), as well as class II promoter activity of CRP in E. coli (29) and the virulence of Salmonella via acetylation (46, 50). Recent reports show that AcP can also acetylate MtrA (51) and NrtR in mycobacteria (45). In the current study, under conditions of AcP accumulation, i.e., using *ackA* mutants with glucose as the single carbon source, the transcription of CRP was repressed and acetylation of K193 was increased (Fig. 6f and g). Therefore, there is a negative correlation between intracellular AcP levels and CRP activity. The acetyltransferase Pat (encoded by *Rv0998*) has acetylase activity that is directly regulated by cAMP binding *in vitro* (52, 53). Due to the negative correlation between cAMP concentration and the acetylation of CRP K193 in mycobacteria (Fig. 4h), we hypothesized that K193 is primarily acetylated nonenzymatically by AcP. Given the importance of CRP in regulating *Mtb* virulence (14), central metabolism (12, 14), and its response to adverse host conditions (31, 32), the role of AcP in *Mtb* physiology should not be underestimated, but it does require additional research. The growth of *Mtb* has been shown to be inhibited after the deletion of *acka* or *pta* in the presence of acetate as the sole carbon source (54). This is also observed in other nonmycobacterial species, such as E. coli (55, 56). This behavior is quite similar to that observed in Msm-CRP^K193R^ and Msm-CRP^K193Q^ mutants (Fig. 3a), suggesting that a substantial part of the *pta* and *ack* mutant effects may be due to the acetylation status of CRP.

Of note, CRP acetylated by AcP can be deacetylated by an NAD^+^-dependent deacetylase (encoded by *Rv1151c*) (Fig. 7a and b). cAMP at 0.01 mM and different stresses significantly increased the expression of Rv1151c in *Mtb* (Fig. 7c and d). Intracellular cAMP concentrations from infected macrophages ranged between 0.26 and 3.2 mM (57), suggesting that the reversible acetylation of *Mtb* can be regulated by the expression of Rv1151c when *Mtb* senses changes to the cAMP concentration in host cells. Therefore, we believe that the decreased fraction of acetylated CRP is due to Rv1151c upregulation and more deacetylase activity when mycobacteria are exposed to the different stresses and increased cAMP (Fig. 4g and h). To be certain, the ability of cAMP to regulate the deacetylase activity of Rv1151c *in vivo* requires further investigation. To the best of our knowledge, this is the first time that the expression of Rv1151c was found to be directly regulated by stresses and cAMP levels, similar to macrophages. Rv1151c had increased expression in the lungs of *Mtb*-infected guinea pigs at 30 and 90 days (58). In the current study, we suggest that cAMP signaling affects protein acetylation modification in *Mtb* not only since CRP is a cAMP-binding protein, as is currently thought, but also through cAMP-dependent upregulation of Rv1151c.

In summary, the current study found that mycobacteria can regulate their pathogenicity through the reversible acetylation of CRP K193. Moreover, this reversible acetylation is mediated by AcP and a NAD^+^-dependent deacetylase. We believe that the complex interconnected regulatory networks among different transcriptional and posttranslational networks are common regulatory mechanisms in prokaryotes. In mycobacteria, the superposition of metabolic intermediates such as cAMP, AcP, and NAD^+^ to this system further complicates the overall global regulatory network.

## MATERIALS AND METHODS

### Bacterial strains.

The CRP K193 mutant strain of M. smegmatis (*Msm*) was constructed using a CRISPR-Cas12a system described by Yan et al. (59). Rv1151c, the NAD^+^-dependent deacetylase found in the *Mtb* genome, is encoded by *Rv1151c* in *Mtb* H37Rv and by *MRA_1161* in H37Ra. H37Ra and its *Rv1151c* deletion (Δ*Rv1151c*) mutant were used in this study (27). E. coli DH5α was used for genetic manipulation of DNA, and E. coli BL21(DE3) was used for expression of the recombinant CRPs and K193 site-specific mutation proteins.

### Expression and purification of recombinant proteins.

For purification of WT and different K193 site-specific mutant CRPs, pET28a-*crp*, pET28a-*crp*K193Q, pET28a-*crp*K193R, and pET28a-*crp*K193A were constructed using the corresponding primers (Table S1). Site-directed mutagenesis of CRP was performed using the KOD-Plus mutagenesis kit according to the manufacturer’s recommendations (Toyobo, Japan) and verified by sequencing. All recombinant plasmids were individually transformed into E. coli strain BL21(DE3) cells, and the resultant strains were grown in LB medium containing 50 μg/mL kanamycin at 37°C.

The expression of CRPs was induced with isopropyl-β-d-1-thiogalactopyranoside (IPTG; 0.4 mM; Sinopharm, Shanghai, China) for 8 h at 25°C. Afterward, cells were harvested by centrifugation and stored at −80°C. The collected bacteria were lysed and then centrifuged at 13,000 rpm for 30 min to clarify the supernatant. The proteins were purified by Ni^2+^ affinity chromatography (Ni Sepharose 6 Fast Flow, Qiagen, Shanghai, China) following the manufacturer’s instructions, which was performed at 4°C. Expression and purification of Rv1151c were performed according to the methods in our previous report (27).

### EMSA.

Electrophoretic mobility shift assays (EMSAs) were carried out to test the binding of DNA probes with CRP and site-mutated proteins, using the LightShift chemiluminescent EMSA kit (Thermo Fisher Scientific, USA). The biotin-labeled target DNA probes, 2D3-F (5′-CTC TAT GTG ACG AAG CCC ACA TCG AC-3′) and 2D3-R (5′- GTC GAT GTG GGC TTC GTC ACA TAG AG-3′), were synthesized by Sangon Biotech (Shanghai, China); they were heated to 95°C for 10 min and then annealed by gradual cooling to 25°C to form double-stranded oligonucleotides. Biotin-labeled DNA, CRP, and reaction components were added in the order recommended by the instructions for the binding reaction at 25°C for 20 min. Following incubation, the entire reaction volume was electrophoresed on a 6% nondenaturing Tris-glycine polyacrylamide gel.

### Generation of homogenous CRP-containing *N*_ε_-acetyllysine at K193.

To generate a recombinant CRP that homogeneously contained acetylated K193, a genetic engineering system was used to direct the site-specific incorporation of *N*_ε_-acetyllysine to the amber codon, as described previously (60). Briefly, the recombinant plasmid pT7-*crp* was constructed, and then the codon of the K193 site was mutated from AAG to TAG to obtain the site-specific mutant recombinant plasmid. This plasmid and two others (pAckRS-3 and pPylT) were then introduced into E. coli Rosetta and plated on LB agar plates containing spectinomycin (150 μg/mL), kanamycin (50 μg/mL), and ampicillin (150 μg/mL). The recombinant E. coli strains containing these three plasmids were grown to log phase (optical density at 600 nm [OD_600_] = 0.6) in LB medium supplemented with the same antibiotics. The culture was then supplemented with IPTG (1 mM), *N*-acetyllysine (2 mM; Sigma-Aldrich, Santa Clara, CA, USA), and NAM (10 mM; Sigma-Aldrich, Santa Clara, CA, USA) and incubated for 10 h at 25°C. CRP acetylated at K193 was purified by Ni^2+^ affinity chromatography at 4°C (Ni Sepharose 6 Fast Flow, Qiagen, Shanghai, China) according to the manufacturer’s instructions.

### Observation of mycobacterial phenotype *in vitro*.

The mutant strains were cultured in 7H9-OADC to log phase, adjusted to an OD_600_ of 0.2, and diluted to 10^−4^ using 10-fold gradients. The resulting bacterial suspension (100 μL) was plated in 7H10-OADC medium and incubated at 37°C for 4 to 8 days. Colony morphology was observed and recorded under a Zeiss Stereo-Discovery V20 microscope.

To evaluate biofilm formation, mycobacteria cultured to log phase were centrifuged at 3,750 rpm for 10 min, washed twice, and resuspended in Sauton’s medium to an OD_600_ of 0.1. The bacterial solution was then diluted 20 times, and the solution (1 mL/well) was added to a 12-well plate in triplicate. After 4 to 8 days of stable incubation in Parafilm-coated 12-well plates, the bacterial biofilms were observed and recorded using a Zeiss Stereo-Discovery V20 microscope.

The morphology of individual bacteria were captured by transmission electron microscopy (TEM; JEM-Z300FSC CRYO ARM 300 microscope; JEOL, Japan). Mycobacteria were cultured in 7H9-OADC to log phase, harvested, and washed three times with phosphate-buffered saline (PBS). Bacteria were then resuspended in fixing reagent (2.5% glutaraldehyde [EM grade; Solarbio, Beijing, China]), stained with 5% uranyl acetate and 3% lead citrate for 15 min, and then dried at 55°C.

### *In vitro* acetylation assay.

The *in vitro* acetylation reaction was carried out by adding CRP (20 μg) to a concentration gradient of AcP in a volume of 100 μL. Reaction mixtures were thoroughly mixed and incubated at 37°C for 2 or 4 h. Specific protein concentrations are indicated in the figure legends.

### *In vitro* deacetylation assay.

Deacetylation of CRP by Rv1151c (encoded by *Rv1151c* in M. tuberculosis) was performed in deacetylation reaction buffer containing Tris-HCl (50 mM; pH 8.0), NaCl (135 mM), KCl (2.5 mM), and MgCl_2_ (1 mM) in the presence or absence of NAD^+^ (1 mM) and in the presence or absence of NAM (10 mM). Specific protein and NAM concentrations are indicated in the figure legends.

### Determination of bacterial resistance to adverse environments.

Msm-CRP^WT^, Msm-CRP^K193R^, and Msm-CRP^K193Q^ strains were cultured to log phase (OD_600_ = 0.6 to 0.8), harvested, resuspended in PBS three times, and then transferred to different conditions in 7H9-OADC medium (0.012% oleic acid, 5% bovine serum albumin [BSA], 2% dextrose, and 5% catalase) including 42°C, 55°C, hypoxia, pH 3.5, pH 4.5, H_2_O_2_ (5 mM), 0.05% SDS, and different concentrations of cAMP, at 37°C (unless stated otherwise) with shaking at 100 rpm. Following treatment, the bacterial solutions were diluted to generate 10-fold gradients, and 100 μL of each was applied to a 7H10-OADC solid plate. The plates were cultured at 37°C for 3 to 4 days, and CFU were counted.

### Preparation of antisera.

Antibodies against the CRP or the acetylated K193 residue of CRP*_Mtb_* were customized by GL Biochem Ltd. (Shanghai, China). To generate the antibodies, purified recombinant CRP and synthesized peptides, including GASRETVN(K-Ac)ALADFAH-Cys and GASRETVNKALADFAH-Cys, were used as antigens to immunize rabbits. The study was approved by the ethics committee at GL Biochem Ltd. Over 2 months, rabbits were immunized four times, and the antiserum was collected. For the specific antibody against K193 acetylation, a control peptide (GASRETVNKALADFAH) was used to remove nonspecific antibody from the anti-K193^Ace^ antisera. The sensitivity and specificity of the antibody were evaluated by enzyme-linked immunosorbent assay (ELISA).

### Western blotting.

Standard Western blot procedures were used in this study. Purified recombinant protein or cell extracts were separated on a 12.5% gel by sodium dodecyl sulfate-polyacrylamide gel electrophoresis and transferred to a polyvinylidene difluoride (PVDF) membrane. The primary antibodies used were anti-CRP (1:2,000) and anti-K193^Ace^ (1:2,000). After incubation with primary antibodies, the membranes were washed and incubated with horseradish peroxidase-conjugated anti-rabbit immunoglobulin secondary antibodies, followed by detection using an enhanced chemiluminescence Western blot substrate (Bio-Rad, USA).

### qPCR assay.

Bacteria were grown to log phase (OD_600_ = 0.6) in 7H9-OADC–0.05% Tween 80 medium (Becton, Dickinson, Sparks, MD, USA) and were harvested and processed by disruption using bead beaters. Total RNA was extracted using an RNAprep Pure Cell/Bacteria kit according to the manufacturer’s recommendations (Tiangen, Shanghai, China). The cDNA was synthesized from 1 μg of total RNA using the HiScript II one-step RT-PCR kit (Dye Plus) (Vazyme, Nanjing, China). qPCR was performed in triplicate, and cDNA was amplified using *Taq* Pro universal SYBR qPCR master mix (Vazyme, Nanjing, China). The relative transcriptional level for each gene was determined according to the 2^−ΔΔ^*^CT^* method, and 16S rRNA was used as the gene of reference. qPCR primers are listed in Table S1.

### Cell infection model.

Mouse macrophage-like RAW264.7 cells were grown at 37°C and 5% CO_2_ in Dulbecco’s modified Eagle medium (DMEM) supplemented with 10% fetal bovine serum (FBS). Cells were seeded at 1 × 10^6^ cells/well in 12-well plates. The bacteria were diluted to achieve an MOI of 20 and then incubated with cells for 4 h at 37°C in 5% CO_2_. After the supernatant medium was discarded and infected cells were washed twice with PBS, the cells were incubated in DMEM with 10% FBS containing gentamicin (100 μg/mL) for 2 h. Infected cells were lysed at the desired postinfection time points using 0.025% SDS. The number of viable intracellular bacteria was determined by serial dilutions and plating. Bacterial growth was measured as the fold change in the number of CFU per milliliter obtained from macrophages between two time points (61).

### Animal studies.

Eight-week-old C57BL/6 female mice were used in this study. Mice were randomly divided into indicated groups (six mice/group). Quantitative (1.0 × 10^8^ CFU bacteria) challenge experiments of Msm-CRP^WT^, Msm-CRP^K193Q^, and Msm-CRP^K193R^ were performed on mice by intravenous injection through the tail vein, and samples were taken at different time points after injection to evaluate the virulence of the three strains in the mouse model. Mice were assessed twice per day for death.

### Histopathology analysis.

Lungs of experimental animals were fixed in 4% neutral buffered paraformaldehyde solution for 24 h. Lung tissue was then embedded in paraffin. A series of sections (5 μm) were stained with hematoxylin and eosin following standard protocols. The pathology was evaluated by experienced pathologists in a blind manner.

### AcP estimation.

AcP was measured as described previously (34, 62, 63). The efficiency of conversion of AcP to ATP is approximately 51 to 55% (62, 64, 65), so the concentration of AcP was estimated by measuring the content of ATP according to the following methods.

The *Mtb* culture was centrifuged at 8,000 rpm for 10 min at 4°C, and the pellet was resuspended in buffer (0.5 mL; 10 mM sodium phosphate [pH 7.5], 10 mM MgCl_2_, 1 mM EDTA). One-quarter volume of precooled 3 M perchloric acid (HClO_4_) was added, and the mixture was incubated on ice for 45 min, followed by centrifugation at 12,000 rpm for 5 min at 4°C. The supernatant was collected and neutralized by the addition of one-quarter volume of saturated KHCO_3_. After centrifugation at 12,000 rpm at 5 and 4°C, activated carbon powder (~7.5 mg per 100 μL lysate) was added, and the mixture was incubated on ice for 15 min to remove endogenous ATP. The carbon powder was removed by rotating filtration through a 0.45 μm PVDF filter (Merck KGaA, Darmstadt, Germany). An ATP conversion assay system (200 μL total; 1 μL 100 mM MgCl_2_, 4 μL 2 mM ADP; Sigma-Aldrich, Santa Clara, CA, USA) and AckA (4 μL; 0.1 μg/μL; Sigma-Aldrich, Santa Clara, CA, USA) were combined, and the mixture was incubated at 30°C for 90 min. ATP was measured by mixing 50 μL conversion mixture with 50 μL CellTiter-Lumi Plus (Beyotime Biotechnology, Shanghai, China) reagent.

### Ethics statement.

Animal procedures were approved by Fudan University School, and this study was carried out in strict accordance with the National Research Council Guide for Care and Use of Laboratory Animals (SYXK [Shanghai 2007–0025]). All surgery was performed under sodium pentobarbital anesthesia, and all efforts were made to minimize suffering.

### Statistical analysis.

Statistical significance was assessed by analysis of variance (ANOVA), and an unpaired two-tailed Student’s *t* test was done using GraphPad Prism 8.0 software; *P* values of <0.05 were considered significant.

## References

[1] Jeremiah C, Petersen E, Nantanda R, Mungai BN, Migliori GB, Amanullah F, Lungu P, Ntoumi F, Kumarasamy N, Maeurer M, Zumla A. 2022. The WHO global tuberculosis 2021 report - not so good news and turning the tide back to end TB. Int J Infect Dis 124:S26–S29. doi:10.1016/j.ijid.2022.03.011. 35321845 PMC8934249

[2] Bai G, Knapp GS, McDonough KA. 2011. Cyclic AMP signalling in mycobacteria: redirecting the conversation with a common currency. Cell Microbiol 13:349–358. doi:10.1111/j.1462-5822.2010.01562.x. 21199259 PMC3785248

[3] Körner H, Sofia HJ, Zumft WG. 2003. Phylogeny of the bacterial superfamily of Crp-Fnr transcription regulators: exploiting the metabolic spectrum by controlling alternative gene programs. FEMS Microbiol Rev 27:559–592. doi:10.1016/S0168-6445(03)00066-4. 14638413

[4] Soberon-Chavez G, Alcaraz LD, Morales E, Ponce-Soto GY, Servin-Gonzalez L. 2017. The transcriptional regulators of the CRP family regulate different essential bacterial functions and can be inherited vertically and horizontally. Front Microbiol 8:959. doi:10.3389/fmicb.2017.00959. 28620358 PMC5449483

[5] McDonough KA, Rodriguez A. 2011. The myriad roles of cyclic AMP in microbial pathogens: from signal to sword. Nat Rev Microbiol 10:27–38. doi:10.1038/nrmicro2688. 22080930 PMC3785115

[6] Agarwal N, Lamichhane G, Gupta R, Nolan S, Bishai WR. 2009. Cyclic AMP intoxication of macrophages by a Mycobacterium tuberculosis adenylate cyclase. Nature 460:98–102. doi:10.1038/nature08123. 19516256

[7] Shenoy AR, Srinivas A, Mahalingam M, Visweswariah SS. 2005. An adenylyl cyclase pseudogene in Mycobacterium tuberculosis has a functional ortholog in Mycobacterium avium. Biochimie 87:557–563. doi:10.1016/j.biochi.2005.01.017. 15908099

[8] Bai G, Schaak DD, McDonough KA. 2009. cAMP levels within Mycobacterium tuberculosis and Mycobacterium bovis BCG increase upon infection of macrophages. FEMS Immunol Med Microbiol 55:68–73. doi:10.1111/j.1574-695X.2008.00500.x. 19076221 PMC3222459

[9] McCue LA, McDonough KA, Lawrence CE. 2000. Functional classification of cNMP-binding proteins and nucleotide cyclases with implications for novel regulatory pathways in Mycobacterium tuberculosis. Genome Res 10:204–219. doi:10.1101/gr.10.2.204. 10673278

[10] Tang WJ, Yan S, Drum CL. 1998. Class III adenylyl cyclases: regulation and underlying mechanisms. Adv Second Messenger Phosphoprotein Res 32:137–151. doi:10.1016/s1040-7952(98)80009-8. 9421589

[11] Spreadbury CL, Pallen MJ, Overton T, Behr MA, Mostowy S, Spiro S, Busby SJW, Cole JA. 2005. Point mutations in the DNA- and cNMP-binding domains of the homologue of the cAMP receptor protein (CRP) in Mycobacterium bovis BCG: implications for the inactivation of a global regulator and strain attenuation. Microbiology (Reading) 151:547–556. doi:10.1099/mic.0.27444-0. 15699203

[12] Bai G, Schaak DD, Smith EA, McDonough KA. 2011. Dysregulation of serine biosynthesis contributes to the growth defect of a Mycobacterium tuberculosis crp mutant. Mol Microbiol 82:180–198. doi:10.1111/j.1365-2958.2011.07806.x. 21902733 PMC3785234

[13] Sassetti CM, Boyd DH, Rubin EJ. 2003. Genes required for mycobacterial growth defined by high density mutagenesis. Mol Microbiol 48:77–84. doi:10.1046/j.1365-2958.2003.03425.x. 12657046

[14] Rickman L, Scott C, Hunt DM, Hutchinson T, Menendez MC, Whalan R, Hinds J, Colston MJ, Green J, Buxton RS. 2005. A member of the cAMP receptor protein family of transcription regulators in Mycobacterium tuberculosis is required for virulence in mice and controls transcription of the rpfA gene coding for a resuscitation promoting factor. Mol Microbiol 56:1274–1286. doi:10.1111/j.1365-2958.2005.04609.x. 15882420 PMC2964915

[15] Agarwal N, Raghunand TR, Bishai WR. 2006. Regulation of the expression of whiB1 in Mycobacterium tuberculosis: role of cAMP receptor protein. Microbiology (Reading) 152:2749–2756. doi:10.1099/mic.0.28924-0. 16946269

[16] Smith LJ, Stapleton MR, Fullstone GJ, Crack JC, Thomson AJ, Le Brun NE, Hunt DM, Harvey E, Adinolfi S, Buxton RS, Green J. 2010. Mycobacterium tuberculosis WhiB1 is an essential DNA-binding protein with a nitric oxide-sensitive iron-sulfur cluster. Biochem J 432:417–427. doi:10.1042/BJ20101440. 20929442 PMC2992795

[17] Knapp GS, Lyubetskaya A, Peterson MW, Gomes AL, Ma Z, Galagan JE, McDonough KA. 2015. Role of intragenic binding of cAMP responsive protein (CRP) in regulation of the succinate dehydrogenase genes Rv0249c-Rv0247c in TB complex mycobacteria. Nucleic Acids Res 43:5377–5393. doi:10.1093/nar/gkv420. 25940627 PMC4477654

[18] Minato Y, Gohl DM, Thiede JM, Chacon JM, Harcombe WR, Maruyama F, Baughn AD. 2019. Genomewide assessment of Mycobacterium tuberculosis conditionally essential metabolic pathways. mSystems 4:e00070-19. doi:10.1128/mSystems.00070-19. 31239393 PMC6593218

[19] Aslebagh R, Wormwood KL, Channaveerappa D, Wetie AGN, Woods AG, Darie CC. 2019. Identification of posttranslational modifications (PTMs) of proteins by mass spectrometry. Adv Exp Med Biol 1140:199–224. doi:10.1007/978-3-030-15950-4_11. 31347049

[20] Walsh CT, Garneau-Tsodikova S, Gatto GJ, Jr. 2005. Protein posttranslational modifications: the chemistry of proteome diversifications. Angew Chem Int Ed Engl 44:7342–7372. doi:10.1002/anie.200501023. 16267872

[21] Kouzarides T. 2000. Acetylation: a regulatory modification to rival phosphorylation? EMBO J 19:1176–1179. doi:10.1093/emboj/19.6.1176. 10716917 PMC305658

[22] Carabetta VJ, Cristea IM. 2017. Regulation, function, and detection of protein acetylation in bacteria. J Bacteriol 199:e00107-17. doi:10.1128/JB.00107-17. 28439035 PMC5527388

[23] Nakayasu ES, Burnet MC, Walukiewicz HE, Wilkins CS, Shukla AK, Brooks S, Plutz MJ, Lee BD, Schilling B, Wolfe AJ, Muller S, Kirby JR, Rao CV, Cort JR, Payne SH. 2017. Ancient regulatory role of lysine acetylation in central metabolism. mBio 8:e01894-17. doi:10.1128/mBio.01894-17. 29184018 PMC5705920

[24] Christensen DG, Baumgartner JT, Xie X, Jew KM, Basisty N, Schilling B, Kuhn ML, Wolfe AJ. 2019. Mechanisms, detection, and relevance of protein acetylation in prokaryotes. mBio 10:e02708-18. doi:10.1128/mBio.02708-18. 30967470 PMC6456759

[25] Bi J, Wang Y, Yu H, Qian X, Wang H, Liu J, Zhang X. 2017. Modulation of central carbon metabolism by acetylation of isocitrate lyase in Mycobacterium tuberculosis. Sci Rep 7:44826. doi:10.1038/srep44826. 28322251 PMC5359664

[26] Yang H, Sha W, Liu Z, Tang T, Liu H, Qin L, Cui Z, Chen J, Liu F, Zheng R, Huang X, Wang J, Feng Y, Ge B. 2018. Lysine acetylation of DosR regulates the hypoxia response of Mycobacterium tuberculosis. Emerg Microbes Infect 7:34. doi:10.1038/s41426-018-0032-2. 29559631 PMC5861037

[27] Bi J, Gou Z, Zhou F, Chen Y, Gan J, Liu J, Wang H, Zhang X. 2018. Acetylation of lysine 182 inhibits the ability of Mycobacterium tuberculosis DosR to bind DNA and regulate gene expression during hypoxia. Emerg Microbes Infect 7:108. doi:10.1038/s41426-018-0112-3. 29899473 PMC5999986

[28] Xie LX, Wang XB, Zeng J, Zhou ML, Duan XK, Li QM, Zhang Z, Luo HP, Pang L, Li W, Liao GJ, Yu X, Li YX, Huang HR, Xie JP. 2015. Proteome-wide lysine acetylation profiling of the human pathogen Mycobacterium tuberculosis. Int J Biochem Cell Biol 59:193–202. doi:10.1016/j.biocel.2014.11.010. 25456444

[29] Davis R, Ecija-Conesa A, Gallego-Jara J, de Diego T, Filippova EV, Kuffel G, Anderson WF, Gibson BW, Schilling B, Canovas M, Wolfe AJ. 2018. An acetylatable lysine controls CRP function in E. coli. Mol Microbiol 107:116–131. doi:10.1111/mmi.13874. 29105190

[30] Bai G, McCue LA, McDonough KA. 2005. Characterization of Mycobacterium tuberculosis Rv3676 (CRPMt), a cyclic AMP receptor protein-like DNA binding protein. J Bacteriol 187:7795–7804. doi:10.1128/JB.187.22.7795-7804.2005. 16267303 PMC1280308

[31] North RJ, Jung YJ. 2004. Immunity to tuberculosis. Annu Rev Immunol 22:599–623. doi:10.1146/annurev.immunol.22.012703.104635. 15032590

[32] Stewart GR, Robertson BD, Young DB. 2003. Tuberculosis: a problem with persistence. Nat Rev Microbiol 1:97–105. doi:10.1038/nrmicro749. 15035039

[33] Abramovitch RB, Rohde KH, Hsu FF, Russell DG. 2011. aprABC: a Mycobacterium tuberculosis complex-specific locus that modulates pH-driven adaptation to the macrophage phagosome. Mol Microbiol 80:678–694. doi:10.1111/j.1365-2958.2011.07601.x. 21401735 PMC3138066

[34] Weinert BT, Iesmantavicius V, Wagner SA, Scholz C, Gummesson B, Beli P, Nystrom T, Choudhary C. 2013. Acetyl-phosphate is a critical determinant of lysine acetylation in E. coli. Mol Cell 51:265–272. doi:10.1016/j.molcel.2013.06.003. 23830618

[35] Kuhn ML, Zemaitaitis B, Hu LI, Sahu A, Sorensen D, Minasov G, Lima BP, Scholle M, Mrksich M, Anderson WF, Gibson BW, Schilling B, Wolfe AJ. 2014. Structural, kinetic and proteomic characterization of acetyl phosphate-dependent bacterial protein acetylation. PLoS One 9:e94816. doi:10.1371/journal.pone.0094816. 24756028 PMC3995681

[36] Wolfe AJ. 2005. The acetate switch. Microbiol Mol Biol Rev 69:12–50. doi:10.1128/MMBR.69.1.12-50.2005. 15755952 PMC1082793

[37] AbouElfetouh A, Kuhn ML, Hu LI, Scholle MD, Sorensen DJ, Sahu AK, Becher D, Antelmann H, Mrksich M, Anderson WF, Gibson BW, Schilling B, Wolfe AJ. 2015. The E. coli sirtuin CobB shows no preference for enzymatic and nonenzymatic lysine acetylation substrate sites. Microbiologyopen 4:66–83. doi:10.1002/mbo3.223. 25417765 PMC4335977

[38] Castano-Cerezo S, Bernal V, Blanco-Catala J, Iborra JL, Canovas M. 2011. cAMP-CRP co-ordinates the expression of the protein acetylation pathway with central metabolism in Escherichia coli. Mol Microbiol 82:1110–1128. doi:10.1111/j.1365-2958.2011.07873.x. 22059728

[39] Ye Q, Ji QQ, Yan W, Yang F, Wang ED. 2017. Acetylation of lysine ϵ-amino groups regulates aminoacyl-tRNA synthetase activity in Escherichia coli. J Biol Chem 292:10709–10722. doi:10.1074/jbc.M116.770826. 28455447 PMC5481575

[40] Zhang Q, Zhou A, Li S, Ni J, Tao J, Lu J, Wan B, Li S, Zhang J, Zhao S, Zhao GP, Shao F, Yao YF. 2016. Reversible lysine acetylation is involved in DNA replication initiation by regulating activities of initiator DnaA in Escherichia coli. Sci Rep 6:30837. doi:10.1038/srep30837. 27484197 PMC4971506

[41] Liarzi O, Barak R, Bronner V, Dines M, Sagi Y, Shainskaya A, Eisenbach M. 2010. Acetylation represses the binding of CheY to its target proteins. Mol Microbiol 76:932–943. doi:10.1111/j.1365-2958.2010.07148.x. 20398208

[42] Baron S, Eisenbach M. 2017. CheY acetylation is required for ordinary adaptation time in Escherichia coli chemotaxis. FEBS Lett 591:1958–1965. doi:10.1002/1873-3468.12699. 28542702

[43] Hu LI, Chi BK, Kuhn ML, Filippova EV, Walker-Peddakotla AJ, Basell K, Becher D, Anderson WF, Antelmann H, Wolfe AJ. 2013. Acetylation of the response regulator RcsB controls transcription from a small RNA promoter. J Bacteriol 195:4174–4186. doi:10.1128/JB.00383-13. 23852870 PMC3754749

[44] Ren J, Sang Y, Tan Y, Tao J, Ni J, Liu S, Fan X, Zhao W, Lu J, Wu W, Yao YF. 2016. Acetylation of lysine 201 inhibits the DNA-binding ability of PhoP to regulate Salmonella virulence. PLoS Pathog 12:e1005458. doi:10.1371/journal.ppat.1005458. 26943369 PMC4778762

[45] Gao R, Wei W, Hassan BH, Li J, Deng J, Feng Y. 2019. A single regulator NrtR controls bacterial NAD (+) homeostasis via its acetylation. Elife 8:e51603. doi:10.7554/eLife.51603. 31596237 PMC6800001

[46] Venkat S, Chen H, Stahman A, Hudson D, McGuire P, Gan Q, Fan C. 2018. Characterizing lysine acetylation of isocitrate dehydrogenase in Escherichia coli. J Mol Biol 430:1901–1911. doi:10.1016/j.jmb.2018.04.031. 29733852 PMC5988991

[47] Venkat S, Gregory C, Sturges J, Gan Q, Fan C. 2017. Studying the lysine acetylation of malate dehydrogenase. J Mol Biol 429:1396–1405. doi:10.1016/j.jmb.2017.03.027. 28366830 PMC5479488

[48] Wang MM, You D, Ye BC. 2017. Site-specific and kinetic characterization of enzymatic and nonenzymatic protein acetylation in bacteria. Sci Rep 7:14790. doi:10.1038/s41598-017-13897-w. 29093482 PMC5665961

[49] Zhou Q, Gomez Hernandez ME, Fernandez-Lima F, Tse-Dinh YC. 2018. Biochemical basis of E. coli topoisomerase I relaxation activity reduction by nonenzymatic lysine acetylation. Int J Mol Sci 19:1439. doi:10.3390/ijms19051439. 29751635 PMC5983628

[50] Ren J, Sang Y, Qin R, Su Y, Cui ZL, Mang ZG, Li H, Lu SY, Zhang J, Cheng S, Liu XY, Li JX, Lu J, Wu WJ, Zhao GP, Shao F, Yao YF. 2019. Metabolic intermediate acetyl phosphate modulates bacterial virulence via acetylation. Emerg Microbes Infect 8:55–69. doi:10.1080/22221751.2018.1558963. 30866760 PMC6455138

[51] Singh KK, Athira PJ, Bhardwaj N, Singh DP, Watson U, Saini DK. 2021. Acetylation of response regulator protein MtrA in M. tuberculosis regulates its repressor activity. Front Microbiol 11:516315. doi:10.3389/fmicb.2020.516315. 33519719 PMC7843721

[52] Nambi S, Basu N, Visweswariah SS. 2010. cAMP-regulated protein lysine acetylases in mycobacteria. J Biol Chem 285:24313–24323. doi:10.1074/jbc.M110.118398. 20507997 PMC2915667

[53] Xu H, Hegde SS, Blanchard JS. 2011. Reversible acetylation and inactivation of Mycobacterium tuberculosis acetyl-CoA synthetase is dependent on cAMP. Biochemistry 50:5883–5892. doi:10.1021/bi200156t. 21627103 PMC3125470

[54] Somashekar BS, Amin AG, Rithner CD, Troudt J, Basaraba R, Izzo A, Crick DC, Chatterjee D. 2011. Metabolic profiling of lung granuloma in Mycobacterium tuberculosis infected guinea pigs: ex vivo 1H magic angle spinning NMR studies. J Proteome Res 10:4186–4195. doi:10.1021/pr2003352. 21732701

[55] Diaz-Ricci JC, Regan L, Bailey JE. 1991. Effect of alteration of the acetic acid synthesis pathway on the fermentation pattern of Escherichia coli. Biotechnol Bioeng 38:1318–1324. doi:10.1002/bit.260381109. 18600733

[56] Schutze A, Benndorf D, Puttker S, Kohrs F, Bettenbrock K. 2020. The impact of ackA, pta, and ackA-pta mutations on growth, gene expression and protein acetylation in Escherichia coli K-12. Front Microbiol 11:233. doi:10.3389/fmicb.2020.00233. 32153530 PMC7047895

[57] Johnson RM, McDonough KA. 2018. Cyclic nucleotide signaling in Mycobacterium tuberculosis: an expanding repertoire. Pathog Dis 76:fty048. doi:10.1093/femspd/fty048. 29905867 PMC6693379

[58] Kruh NA, Troudt J, Izzo A, Prenni J, Dobos KM. 2010. Portrait of a pathogen: the Mycobacterium tuberculosis proteome in vivo. PLoS One 5:e13938. doi:10.1371/journal.pone.0013938. 21085642 PMC2978697

[59] Yan MY, Yan HQ, Ren GX, Zhao JP, Guo XP, Sun YC. 2017. CRISPR-Cas12a-assisted recombineering in bacteria. Appl Environ Microbiol 83:e00947-17. doi:10.1128/AEM.00947-17. 28646112 PMC5561284

[60] Neumann H, Peak-Chew SY, Chin JW. 2008. Genetically encoding N (epsilon)-acetyllysine in recombinant proteins. Nat Chem Biol 4:232–234. doi:10.1038/nchembio.73. 18278036

[61] Mulder DT, Cooper CA, Coombes BK. 2012. Type VI secretion system-associated gene clusters contribute to pathogenesis of Salmonella enterica serovar Typhimurium. Infect Immun 80:1996–2007. doi:10.1128/IAI.06205-11. 22493086 PMC3370595

[62] Sharma S, Kumari P, Vashist A, Kumar C, Nandi M, Tyagi JS. 2019. Cognate sensor kinase-independent activation of Mycobacterium tuberculosis response regulator DevR (DosR) by acetyl phosphate: implications in anti-mycobacterial drug design. Mol Microbiol 111:1182–1194. doi:10.1111/mmi.14196. 30589958

[63] Pruss BM, Wolfe AJ. 1994. Regulation of acetyl phosphate synthesis and degradation, and the control of flagellar expression in Escherichia coli. Mol Microbiol 12:973–984. doi:10.1111/j.1365-2958.1994.tb01085.x. 7934904

[64] Guerlava P, Izac V, Tholozan JL. 1998. Comparison of different methods of cell lysis and protein measurements in Clostridium perfringens: application to the cell volume determination. Curr Microbiol 36:131–135. doi:10.1007/pl00006756. 9516540

[65] Schaechter M. The View From Here Group. 2001. Escherichia coli and Salmonella 2000: the view from here. Microbiol Mol Biol Rev 65:119–130. doi:10.1128/MMBR.65.1.119-130.2001. 11238988 PMC99021

