# Supplementary material for: Acetylation of Cyclic AMP Receptor Protein by Acetyl Phosphate Modulates Mycobacterial Virulence
Source: Microbiol Spectr. 2023 Jan 26;11(1):e04002-22. doi: 10.1128/spectrum.04002-22 (PMC9927398; doi:10.1128/spectrum.04002-22)
Supplement: Supplemental file 1 — Supplemental material. Download spectrum.04002-22-s0001.pdf, PDF file, 0.6 MB [file spectrum.04002-22-s0001.pdf]

Acetylation of cyclic adenosine monophosphate receptor protein by acetyl phosphate modulates mycobacteria virulence

Running title:

AcP-mediated CRP acetylation modulates mycobacteria virulence

Authors:

Yuchang Di[1], Siyue Xu[1], Mingzhe Chi[1], Youwei Hu[1], Xiao Zhang[1] Honghai Wang[1], Wenhong Zhang[* 1 2], Xuelian Zhang[* 1]

Table S1. Primers used in this study

Primer	Sequence（5’ -3’ ）
pT7-Rv3676-C-F	CGCCATATGGTGGACGAGATCCTGGCCAGGGCAG
pT7-Rv3676-C-R	CCCAAGCTTTTACATCACCATCATCACCACCCTCGCTCG GCGGGCCAGT
193Q-F	AACCAGGCACTGGCTGATTTCGCTCACCGCGGCTG
193R-F	AACAGGGCACTGGCTGATTTCGCTCACCGCGGCTG
193A-F	AACGCGGCACTGGCTGATTTCGCTCACCGCGGCTG
193-R	CACCGTCTCGCGTGAGGCCCCGACCAGCT
pT7-crp-ATG-R	TAGGCACTGGCTGATTTCGCTCACC
pT7-crp-ATG-R	GTTCACCGTCTCGCGTGAGGCCCCG
vivto-test-F	AGCGTTGGCACGGCGAACCGGTT
vivto-test-R	GCAGCCGCACAATCGCACAAAATCC
bing-DNA -F	CTCTATGTGACGAAGCCCACATCGAC
bing-DNA-R	GTCGATGTGGGCTTCGTCACATAGAG
6189-crRNA-F	ATTTGACGGTCTCGCGCGACGCGCCGAA
6189-crRNA-R	AGCTTTCGGCGCGTCGCGCGAGACCGTCAAATCT
6189-K193Q- ssDNA	TCGGCGCGTCGCGCGAGACCGTCAACCAGGCGCTGGCC GACTTCGCCCACCGCGG
6189-K193R-	TCGGCGCGTCGCGCGAGACCGTCAACCGCGCGCTGGCC

ssDNA	GACTTCGCCCACCGCGG
6189-K193A-	TCGGCGCGTCGCGCGAGACCGTCAACGCCGCGCTGGCC
ssDNA	GACTTCGCCCACCGCGG
6188-qPCR-F	TGTCTTGGCTGCTGGTGG
6188-qPCR-R	GTAGATGGGCGTCGGAAGAG
6189-qPCR-F	ATCTTCCAGGGAGTCGAACC
6189-qPCR-R	CGATCTCTTCCTGCGTGAGG
6190-qPCR-F	TGGTGCTCATCAGCCACAAG
6190-qPCR-R	TCGGTGTAGACGTGTTCGAC
0929-qPCR-F	AGGTCAGCGGTCAATTCCAG
0929-qPCR-R	AACTCGCCGTTGTTGGAGAT
5700-qPCR-F	GCCACCAAGGAAGAGCAGAT
5700-qPCR-R	GAGGCGTCGACGATCTGG
3197-qPCR-F	CCGATCGTGTCCTACCAGTG
3197-qPCR-R	TGGTGCTCATCTTGTGCAGG
3233-qPCR-F	TCATCATCTCGGCGAACTCG
3233-qPCR-R	CCCGAACTTCTCCTCGTAGC
4891-qPCR-F	ACGATCGGTGACCAGTTTCC
4891-qPCR-R	GGATCGCCCTTCTTCCAGTT
1919-qPCR-F	GTCTGTCGCGACGAGGATC

Supplementary Figure legends

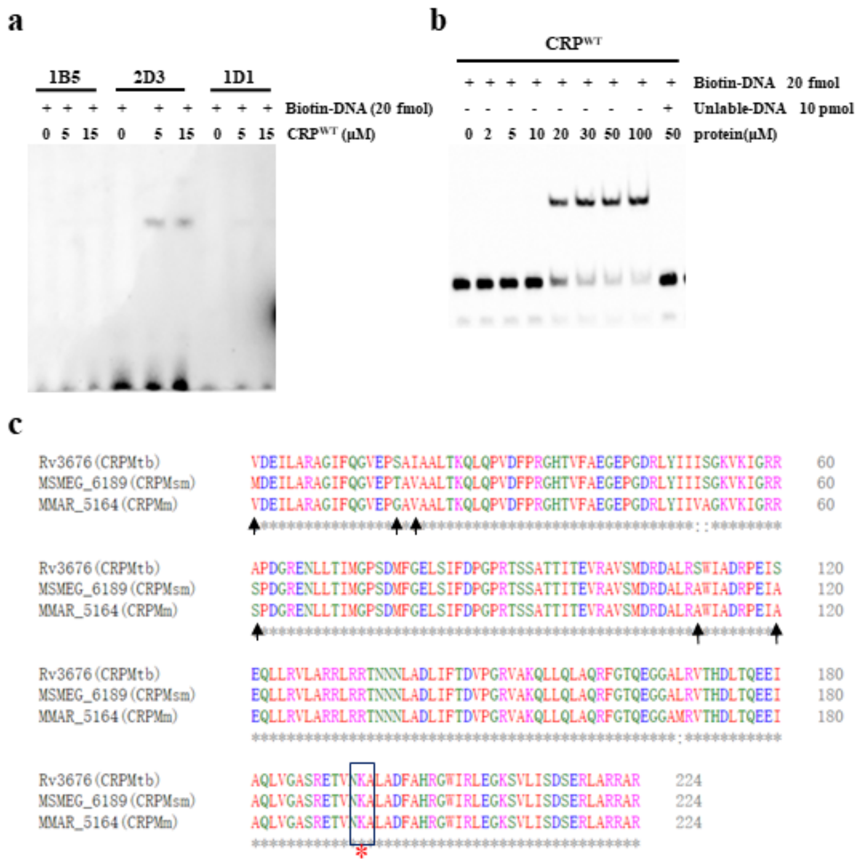

Fig. S1. **CRP protein can bind target DNA, and cAMP enhances its DNA-binding affinity**. (a) Three target DNA sequences of the CRP protein determined previously were selected to incubate with CRP for EMSA. Panel from left to right: lane 1-3: target DNA 1B5; lane 4-6: target DNA 2D3; lane 7-9: target DNA 1D1. (b) DNA-binding abilities of CRP^{WT} by EMSA. EMSA was used to test the binding of the indicated concentrations of CRP (lanes 2 to 9) to Biotin-labeled target DNA 2D3. Lane 1 represents the labeled DNA alone. (c) Conservation analysis of CRP K201 of mycobacteria through sequence alignment. The black arrows denote the six different amino acids between CRP_{Mtb} and CRP_{Msm}, and the red asterisk denotes the conserved lysine residue (K193). The result was

analyzed by BioEdit 7.0. EMSA results are representative of at least three independent replicates. Abbreviations: CRP, cAMP receptor protein; cAMP, cyclic adenosine monophosphate; EMSA, electrophoretic mobility shift assay; WT, wild-type; *Msm*, *Mycobacterium smegmatis*; *Mtb*, *M. tuberculosis*.

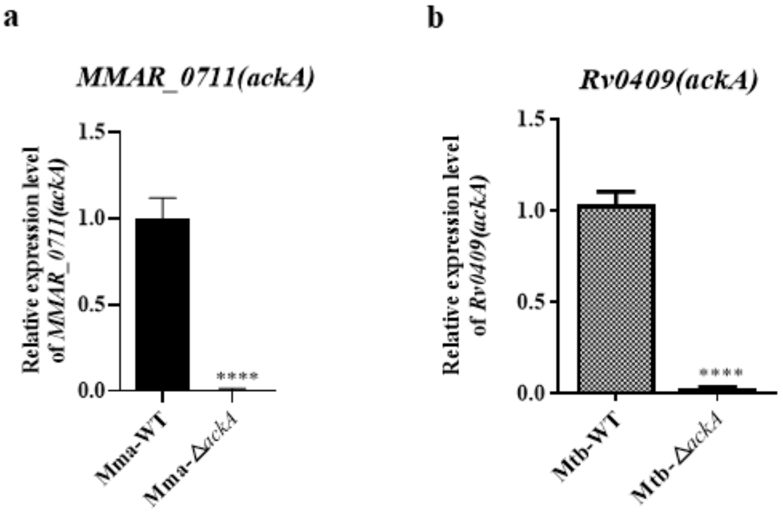

Fig. S2. ***ackA*-knockout strain was successfully constructed by transcriptional level verification in *Mma* and *Mtb***. The transcript levels of *MMAR_0711* (*ackA*) (a) and *Rv0409* (*ackA*) (b) in *ackA*-knockout strains, respectively. Bacteria were grown to OD_{600}=0.6 and harvested to isolate total RNA. The transcript levels were determined by qPCR and $2^{-\Delta\Delta Ct}$ methods. The expression of the tested gene was normalized to that of 16S rRNA and compared to the wild-type strain. *, P<0.05; **, P<0.01, ANOVA analysis. Abbreviations: ackA, acetate kinase; *Mma*, *Mycobacterium marine*; *Mtb*, *M. tuberculosis*; qPCR, quantitative polymerase chain reaction; ANOVA, analysis of variance.

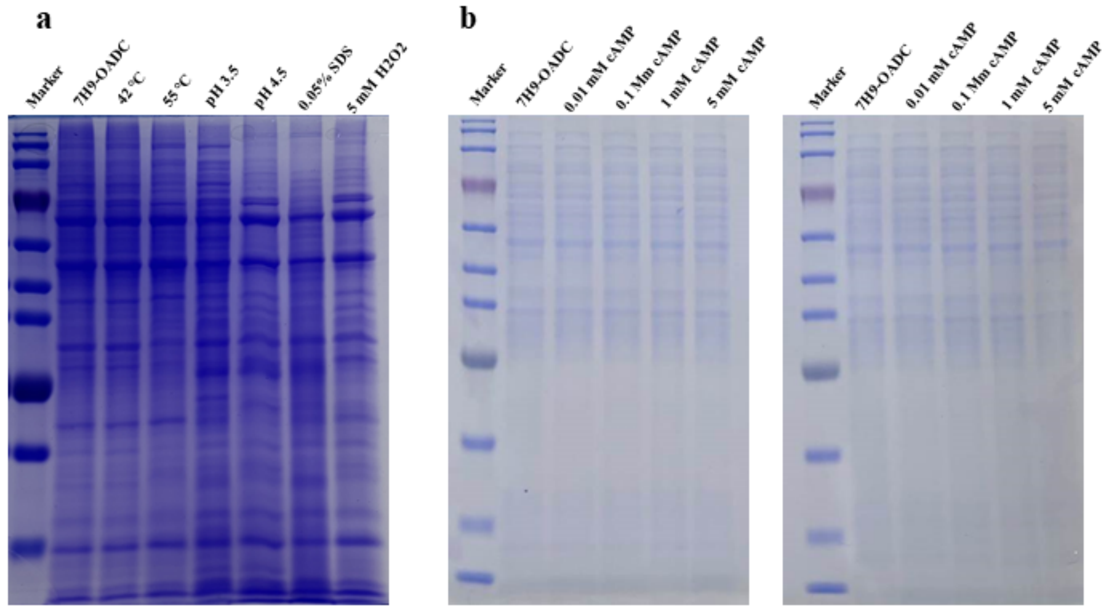

Fig. S3. **Loading control for western blotting analysis**. (a) Loading control for Fig. 4g (a), Fig. 4h (b), and Fig. 7c (c). The strains exposed to different conditions were collected by centrifugation. Cell extracts (20 μg per lane) was analyzed by SDS-PAGE and stained with Coomassie blue. Abbreviations: SDS-PAGE, sodium dodecyl-sulfate polyacrylamide gel electrophoresis.
